# Anti-Inflammatory, Antithrombotic and Antioxidant Efficacy and Synergy of a High-Dose Vitamin C Supplement Enriched with a Low Dose of Bioflavonoids; In Vitro Assessment and In Vivo Evaluation Through a Clinical Study in Healthy Subjects

**DOI:** 10.3390/nu17162643

**Published:** 2025-08-14

**Authors:** Vasiliki Chrysikopoulou, Aikaterini Rampaouni, Eleni Koutsia, Anna Ofrydopoulou, Nikolaos Mittas, Alexandros Tsoupras

**Affiliations:** Hephaestus Laboratory, School of Chemistry, Faculty of Sciences, Kavala University Campus, Democritus University of Thrace, St. Lucas, 65404 Kavala, Greece; vgchrys@chem.duth.gr (V.C.); airabao@chem.duth.gr (A.R.); elkoutd@chem.duth.gr (E.K.); anofrid@chem.duth.gr (A.O.); nmittas@chem.duth.gr (N.M.)

**Keywords:** vitamin C, flavonoids, bioflavonoids, chronic inflammation, antioxidant activity, anti-inflammatory, synergistic interaction, phenolic compounds, cancer prevention, cardiovascular disease

## Abstract

Background/Objectives: Vitamin C is frequently used in several dietary supplements due to its proposed health-promoting properties, while phenolic compounds and especially flavonoids have been suggested to provide synergistic antioxidant and cardiovascular benefits. However, the specific interactions between these compounds and their individual contributions to biological activity remain underexplored. This study aimed to evaluate the antioxidant potential and anti-inflammatory and antiplatelet biological effects of a high-dose (1 g) vitamin C–low-dose (50 mg) bioflavonoid (VCF)-based supplement using both in vitro and in vivo approaches in human platelets. Methods: Total phenolic content was quantified and antioxidant capacity was assessed using DPPH, FRAP, and ABTS assays and compared to individual phenolic standard compounds, including (simple phenolics like gallic acid, flavonoids like quercetin and catechin, and polyphenols like curcumin and tannin), and a standard supplement containing only high-dose vitamin C (VC). ATR-FTIR spectroscopy was used to assess molecular interactions between vitamin C and flavonoids. In vitro anti-inflammatory and antiplatelet activities of all supplements and standards were assessed by quantifying their IC_50_ values against ADP, PAF, and thrombin-induced platelet aggregation. The in vivo evaluation of the efficacy and synergy of VCF supplement versus VC was achieved by a two-arm clinical study in healthy volunteers by quantifying their platelet reactivity, which was measured via EC_50_ values on the aforementioned platelet agonists (PAF, ADP, and Thrombin) before (t = 0) and after receiving either solely VC or VCF supplementation for four weeks. Results: From all phenolic standards, the flavonoids and especially a mixture of flavonoids (catechin + quercetin) showed higher in vitro antioxidant capacity and anti-inflammatory and antiplatelet efficacy, followed by polyphenols and then simple phenolics. The VCF supplement showed the most potent antioxidant capacity, but also the strongest anti-inflammatory and antiplatelet activities too, in comparison to the VC and the mixture of flavonoids, suggesting higher synergy and thus bio-efficacy as a result of the co-presence of flavonoids and vitamin C in this supplement. Platelet reactivity decreased over time for PAF and thrombin in both arms of the trial, but no significant differences were observed between treatment groups, suggesting that the number of flavonoids used was not sufficient to translate the in vitro findings to the in vivo setting. Conclusions: VC-containing supplements provide antioxidant, anti-inflammatory, and antiplatelet benefits, while the incorporation of flavonoids may provide synergistic health benefits, but more in vivo assessment is needed to fully evaluate the dose efficacy.

## 1. Introduction

Inflammation is a physiological, tightly regulated, and protective response to infectious or non-infectious stimuli, involving cells of the innate and adaptive immune systems and inflammatory mediators; however, when it becomes excessive, self-directed, or fails to resolve, it contributes to the pathology of numerous diseases [1]. Chronic inflammatory diseases span a broad spectrum of conditions that collectively impact millions worldwide. For instance, atherosclerosis is now widely recognized as a chronic inflammatory disease, where immune cell dysregulation, vascular smooth muscle cell phenotypic switching, and pro-inflammatory mediators like platelet-activating factor (PAF) play critical roles in plaque formation and cardiovascular complications [2,3,4,5].

In this type of inflammatory response, there are involved biomolecules that enhance inflammation, and, mostly, platelet activation, leading to a cycle of thrombosis and inflammation and thus act as a substrate for chronic diseases [3,4,5]. More specifically, there are three agonists, platelet activation factor (PAF), adenosine diphosphate (ADP), and thrombin, which act in three different pathways, leading to either solely inflammatory (PAF) or thrombo-inflammatory (PAF, ADP or thrombin) response enhancement. PAF is a phospholipid mediator, produced from different types of cells. Its connection with his receptor, PAF-R, leads to activation of pathways that focus on calcium concentration increase in the cells, as well as activation of phospholipase A2 and release of thromboxane A2 [5]. Thus, there is continuous activation of platelets, which can lead to endothelial dysfunctions, atherothrombosis, and ultimately metastasis and angiogenesis [4]. Thrombin is a serine protease, playing a crucial role in hemostasis and also playing a major role in platelet activation with two receptors; this enhances the last step of platelet accumulation and relates thrombin to cancer and metastasis [6,7]. Finally, adenosine diphosphate (ADP) is released from activated platelets, thereby amplifying the platelet activation cascade and promoting further aggregation [8]. More specifically, ADP plays a pivotal role in promoting thrombosis, and elevated levels of ADP are a common feature in various pathological conditions, including atherothrombosis and related cardiovascular diseases [7]. The combined activity of these three agonists appears to play a pivotal role in the regulation of inflammation and thrombosis, thereby contributing to the pathophysiology of inflammation-related disorders, including cancer [5].

Cancer emerges as a leading cause of mortality, with the rising incidence across multiple cancer types and persistent disparities—especially among younger adults and underserved populations—underscoring the urgent need for improved prevention strategies and equitable access to care [9,10]. Conventional therapies for many diseases are often limited by factors such as high costs and adverse long-term side effects, largely attributable to the nature of synthetic chemical agents. Consequently, there has been growing scientific interest in exploring the therapeutic potential of naturally derived compounds for the treatment of various inflammatory diseases. Several dietary bioactives have been proposed as possessing anti-inflammatory, antioxidant, and antithrombotic potency, with antioxidants, vitamins like vitamin C, and flavonoids being the most promising health-promoting natural bioactive compounds for use against oxidative stress, inflammation, and related disorders, including cancer [11,12,13].

Vitamin C, or Ascorbic Acid, is a water-soluble organic compound that belongs to a class of unsaturated polyhydroxy alcohols, structurally defined by a five-membered lactone ring with multiple hydroxyl groups. Its distinctive double bonds between carbon atoms C2 and C3 give its strong reducing and antioxidant properties [14]. The recommended daily intake of vitamin C is 90 mg for adult men and 75 mg for adult women. Nevertheless, since first suggested by Linus Pauling, a Nobel laureate, it has been proposed that daily doses significantly higher than the generally recommended amount of vitamin C can prevent and treat not only the common cold but can also exhibit other health benefits. While his claims have been controversial and not always supported by scientific consensus, his work spurred significant public interest and research into vitamin C’s potential roles in health.

For example, vitamin C has been reported to exhibit a dual role as both an antioxidant and prooxidant in low and high concentrations, respectively [15]. Specifically, one study showed that in septic organ injury, it reduced oxidative stress by modulating markers like Malondialdehyde (MDA), Superoxide Dismutase (SOD), and Glutathione Peroxidase (GSH-Px), and suppressed Reactive Oxygen Species (ROS) generation [16], while, in osteosarcoma cells, high-dose ascorbate promoted ROS production via the Fenton reaction, disrupting mitochondrial function and leading to cell death [17].

Aside from its oxidative properties, vitamin C has also shown great anti-inflammatory capabilities by reducing various inflammatory markers, including Macrophage Inflammatory Protein 1 (MIP-1), Interleukin-10 (IL-10), lymphotactin, Interleukin-1 (IL-1), Bone Morphogenetic Protein (BMP), and Insulin-like Growth Factor (IGF), as well as eotaxin, Monocyte Chemoattractant Protein-1 (MCP-1), Thymus and Activation-Regulated Chemokine (TARC), and Transforming Growth Factor Beta (TGF-β) isoforms [18]. In cancer-related in vivo studies, it enhanced immune responses by increasing Cluster of Differentiation 8 Positive T (CD8+ T) cell infiltration, IL-2 secretion, and tumor cell killing [19]. Additionally, it exhibits antithrombotic activity, particularly against PAF-induced platelet activation, likely due to its ability to counteract ROS, with stronger effects observed in non-oxidized forms compared to oxidized or supplemental forms [20]. These highlight the immense potential of vitamin C as a treatment.

Several studies have demonstrated that vitamin C, widely recognized as the most commonly used hydrophilic antioxidant, possesses several other chronic inflammatory manifestations. For example, vitamin C supports physiological function and aids recovery in HIV-infected individuals by mitigating oxidative stress, particularly when administered in conjunction with Highly Active Antiretroviral Therapy (HAART) [21]. Similarly, vitamin C has demonstrated potential in preserving mitochondrial function and mitigating sepsis-induced organ damage due to reactive oxygen species (ROS) and dysregulated inflammatory response to infection [22].

Phenolic compounds are mainly found as naturally occurring secondary metabolites of plants, and that is why they are usually referred to as phytochemicals too. Structurally, phenolic compounds are characterized by the presence of one or more hydroxyl groups directly attached to an aromatic ring. The term ‘phenolic’ originates from phenol, the simplest representative of this chemical class. Based on their structural complexity and functional groups, phenolic compounds can be broadly categorized into three major groups: simple phenolics, flavonoids, and polyphenolic compounds (polyphenols). Each group exhibits distinct chemical properties and biological activities that contribute to their different roles in plant physiology and human health [23,24,25].

Simple phenolic compounds like gallic acid, thymol, and vanillin consist of a single phenolic ring with one or more hydroxyl groups attached, usually possessing distinct aromas and strong flavors—for example, vanillin, the primary component of vanilla, and thymol, which is derived from the essential oils of thyme. Beyond their use in the food industry as additives to enhance sensory qualities such as aroma, taste, and color, simple phenolics have garnered considerable pharmacological interest. They are recognized for their natural antimicrobial, antioxidant, and anti-inflammatory properties and have demonstrated therapeutic potential against neurological disorders and various cancers [26,27,28,29,30]. Polyphenols, in contrast, are molecules of usually high molecular weight consisting of more than two aromatic rings to which hydroxyl groups are attached, with representative compounds being tannin and tannic acid, as well as curcumin, which is the simplest polyphenol as it is a dimeric phenolic compound. Tea, wine, fruit, vegetables, and various edible plants contain large quantities of these polyphenolic molecules, while their importance lies in their role in the prevention of diseases such as various types of malignancies, cardiovascular diseases, neurodegenerative diseases and disorders such as diabetes, and atherosclerosis due to their anti-inflammatory, antimicrobial, antiplatelet, antithrombotic, and antioxidant properties [31,32,33].

Nevertheless, the most active group of phenolic compounds has been proposed to be the flavonoids, often referred to as Bioflavonoids [13]. They are a widely distributed group, mainly produced and found in plant-based sources, including vegetables, grains, fruits, grains, and herbs but also in many beverages like tea, wine or juices, being responsible for their vibrant colors, flavor or pharmacological activities [34,35,36]. Structurally, flavonoids are characterized by their possession of fifteen carbon atoms arranged with two benzene rings and a heterocyclic ring with oxygen between them as a connection. Flavonoids can be further classified based on their structure and oxidation state into flavones, isoflavones, flavonols, flavanols, anthocyanidins, and flavans in relation to their structure and oxidation state [34,37]. Similarly, in natural sources, they are classified as aglycones or in conjugated forms like flavonols or tannins and proanthocyanidins.

Due to their great potential in health-promoting effects, they have gained attention as bioactive molecules with anti-inflammatory, antioxidant, antitumor, cardioprotective, and cancer-related activities [34,37,38,39]. Historically, dating back to Ancient Greece, flavonoids have been used for their healing properties, as reported by Hippocrates, the father of modern medicine who used propolis for sore and ulcer treatment [40]. Many flavonoids are integral to organismal defense and various biological processes, while they interact with key regulatory pathways involved in apoptosis and immune responses. Additionally, flavonoids have been shown to modulate several cancer types, including leukemia, breast cancer, and bladder cancer [13]. More specifically, flavonoids are able to inhibit cancer metastasis and reduce tumor growth, targeting very critical stages in carcinogenesis [34]. Moreover, flavonoids are known to influence reactive oxygen species (ROS), which play a dual role through inhibiting tumor growth and contributing to carcinogenesis [41].

However, flavonoids are facing multiple limitations regarding their bioavailability and their administration, since their characteristics exhibit low solubility and absorption along with poor intrinsic transmembrane diffusion and metabolism. Due to these limitations, their therapeutic potential is still being researched with synergistic pathways to increase their bioavailability or with systemic and local administration [39,42]. Therefore, our interest in these popular natural-based sources has led us to detect specific synergistic interactions between flavonoids and vitamin C in order to enhance the multiple health benefits that they both have.

For example, the co-presence of citrus flavonoids with vitamin C has been proposed to enhance the bioactivities of both classes of compounds [20], while recently *Rosa rubiginosa* L. leaf extracts rich in such phenolic bioactives have demonstrated strong scavenging activity and high phenolic content protection while also preventing DNA breakage, with protective effects on human primary keratinocytes and fibroblasts due to its strong antioxidant and anti-inflammatory response under oxidative stress induced by UVA irradiation [43]. Quercetin has shown synergistic effects improvement in combination with vitamin C in cancer cells. Specifically, the combination of vitamin C and quercetin resulted inducing Nuclear Factor Erythroid 2-Related Factor 2 (Nrf2) mediated oxidative stress, enhancing total antioxidant activity while reducing cancer cell viability [44]. Moreover, quercetin treatment has exhibited enhancement of anticancer effects of vitamin C via inhibition of Protein Kinase B (Akt) and Mechanistic Target of Rapamycin (mTOR) pathways, while increasing apoptosis in cancer cells with caspase-3 activation [45]. Furthermore, hawthorn extract, which is rich in flavonoids, synergistically enhanced the antioxidant activity of vitamin C along with oxidative stress prevention, normalization of arterial walls, and hypertension mitigation in vivo in rats [46]. Thus, hawthorn extract and vitamin C showed improvement innitric oxide bioavailability, lipid peroxidation reduction, and anti-inflammatory effects.

The present study aims to investigate the antioxidant, anti-inflammatory, and antiplatelet activities of a combined vitamin C and bioflavonoid food supplement, a vitamin C supplement alone, and selected representative phenolic compound standards using clinical trials alongside ex vivo and in vitro models. More specifically, we initially screened in vitro several phenolic standard compounds (simple phenolics, flavonoids, and polyphenols), shown in Table 1, for their potential antioxidant and anti-inflammatory capacity in order to evaluate if indeed the flavonoids possess the strongest potency against inflammation, thrombosis, and oxidative stress. Then, we evaluated the modulatory effects of the presence of naturally derived supplements containing primarily vitamin C against mediators of inflammation and thrombosis, including PAF, ADP, and thrombin, and we also evaluated whether these effects potentially enhanced the bio-efficacy of such supplements to attenuate the pro-inflammatory and pro-thrombotic actions of these pro-inflammatory and pro-thrombotic agonists via the synergy of bioactive citrus and rose-derived flavonoids with vitamin C.

This research aims to investigate the potential synergistic effects and interactions among these compounds, elucidate their mechanisms of action, and assess their overall bioactivity. These findings support the potential role of dietary supplements as antiplatelet and anti-inflammatory agents with both preventive and therapeutic implications for thrombo-inflammatory diseases. Ultimately, our aim is to contribute to the development of natural-based therapeutic strategies for the prevention and management of chronic inflammatory diseases, including cancer and cardiovascular disorders.

## 2. Patients, Materials and Methods

### 2.1. Materials, Reagents, and Instrumentation

Vitamin C and bioflavonoid supplements, as well as vitamin C-only supplements, were purchased from a local pharmacy in Kavala, Greece. All reagents used in the study—including Folin–Ciocalteu reagent, 1,1-diphenyl-2-picrylhydrazyl (DPPH), 2,2′-azinobis-(3-ethylbenzothiazoline-6-sulfonic acid) (ABTS), and solvents such as physiological saline—along with standard phenolics (Trolox) were obtained from Sigma-Aldrich (St. Louis, MO, USA).

UV-Vis spectroscopic analyses were conducted using an LLG-uniSPEC 2 spectrophotometer, while ATR-FTIR spectroscopy was performed on a PerkinElmer Frontier ATR/FT-NIM/MIR spectrometer. For antiplatelet assays, all plastic consumables, reagents, and solvents were of analytical grade and purchased from Sigma-Aldrich. Blood sampling was carried out using 20-gauge safety needles and evacuated sodium citrate S-monovettes acquired from Sarstedt Ltd. (Wexford, Ireland).

Bioassays on human platelet-rich plasma (hPRP) were performed using a quadruple-channel strobilometric platelet aggregometer, Chrono-log 490 (Havertown, PA, USA), equipped with the AGGRO/LINK ^®^8 software package. All consumables for platelet aggregometry were sourced from Chrono-log (Havertown, PA, USA). Standards of platelet-activating factor (PAF), adenosine diphosphate (ADP), and bovine serum albumin (BSA) were also purchased from Sigma-Aldrich. Centrifugation procedures were conducted using a Nahita Blue Medimas centrifuge with a maximum speed of 4000 rpm.

### 2.2. Preparation of Supplement Samples for In Vitro Assessment

To ensure repeatability and enable statistical analysis, all experiments were conducted using triplicate samples of each vitamin tablet. Each tablet was dissolved in 10 mL of physiological saline and stored in a closed, dark environment until complete dissolution to prepare the stock solution for ex vivo assessments (Stock A). For in vitro experiments, tablets were similarly dissolved in 10 mL of deionized water to prepare Stock B. All in vitro assays were performed in triplicate to maintain consistency and reliability of the results.

#### Preparation of Phenolic Standard Samples for In Vitro Assessment

For the antiplatelet and antithrombotic assessments, 30 mg of bovine serum albumin (BSA) was dissolved in 12 mL of physiological saline (0.9% NaCl). This BSA solution was used as the solvent to dissolve all standard compounds tested. Each compound was accurately weighed using an analytical balance and transferred into dry test tubes before being dissolved in the BSA solution. The quantities of each compound and the corresponding volume of BSA were adjusted to achieve a uniform concentration of 10 mg/L for all tested standards.

For antioxidant evaluations, standard substances, including curcumin, thymol, quercetin, catechin, vanillin, tannin, tannic acid, and gallic acid, were precisely weighed on an analytical balance and transferred to dry test tubes. Methanol was added using a calibrated syringe to prepare solutions of known concentrations (expressed in mg/mL). The test tubes were vortexed for 20–25 s to ensure complete dissolution of each compound. Separate solutions were prepared for each antioxidant assay (FRAP, ABTS, DPPH), resulting in varying concentrations across tests. All measurements were conducted within 24 h of preparation to minimize compound degradation. The quantities of compounds and volumes of methanol used were calculated to yield final concentrations close to 10 mg/L for each assay.

### 2.3. Total Phenolic Content Analysis

The total phenolic content of the supplements was determined using the Folin–Ciocalteu reagent method, adapted from [47] with minor modifications. Briefly, one tablet was dissolved in 10 mL of distilled water to prepare the initial stock solution. A 1:10 dilution was then performed by mixing 0.5 mL of this stock with 4.5 mL of distilled water. Subsequently, 1 mL of distilled water and 1 mL of Folin–Ciocalteu reagent were added to the diluted sample. After a 7 min incubation, 3 mL of sodium carbonate (Na_2_CO_3_) solution was added, and the mixture was incubated in the dark for 2 h. The samples were vortexed immediately after each reagent addition and every 30 min during incubation to ensure thorough mixing. Following incubation, absorbance was measured at 765 nm using a UV-Vis spectrophotometer. The total phenolic content was quantified based on a gallic acid standard curve and expressed as mg gallic acid equivalents (GAE) per gram of sample.

### 2.4. Assessment of Antioxidant Activities of Supplements

To assess the antioxidant activity of the supplements, three bioassays were employed: the DPPH radical scavenging assay, the ABTS radical cation decolorization method, and the ferric-reducing antioxidant power (FRAP) assay, following the protocol described in [47] with some modifications.

For all assays, 0.1 mL of Stock B was diluted with 9.9 mL of deionized water. From this solution, serial dilutions were prepared at 1:10, 1:100, and 1:1000, yielding three sample concentrations.

#### 2.4.1. DPPH Assay

For each dilution, 0.2 mL of sample was mixed with 0.8 mL deionized water and 1 mL of DPPH solution. Samples were vortexed after each reagent addition and incubated in the dark at room temperature for 30 min. Absorbance was then measured at 517 nm. A blank solution containing 0.2 mL ethanol, 0.8 mL water, and 1 mL DPPH solution was prepared and measured under the same conditions. The percentage of DPPH radical inhibition was calculated as follows:

Inhibition (%) = {(A1 − A2) × 00}/A1 w(1)

where A1 is the absorbance of our sample solution and A2 the absorbance of the test sample solution. Afterwards, IC_50_ (the concentration of each sample which is able to neutralize 50% of DPPH radicals) was calculated and the DPPH radical scavenging activity of the sample was expressed as Trolox equivalent antioxidant capacity (TEAC) using the following equation:

TEAC = IC_50_ of Trolox (μg/L)/IC_50_ of sample (μg/L) (2)

#### 2.4.2. ABTS Assay

For the ABTS assay, 2 mL of sample solution was mixed with 2 mL of ABTS reagent, vortexed, and incubated in the dark for 7 min. Absorbance was measured at 734 nm, with deionized water used as the blank. Trolox was used as the standard, with absorbance values ranging from 0.2 to 0.8 to construct the standard curve. Results were expressed as μmol Trolox equivalents per gram dry weight (μmol TE/g DW) calculated by the following:

ABTS (μmol TE/g DW) = C × V × t/m(3)

where C represents the Trolox concentration obtained from the standard curve of the diluted sample, V is the volume of the sample, t is the diluting factor, and m the weight of the sample.

#### 2.4.3. FRAP Assay

For the FRAP assay, 3 mL of FRAP reagent was added to each diluted sample, vortexed, and incubated in the dark at room temperature for 15 min. Absorbance was measured at 593 nm. Trolox was again used as a standard with concentrations ranging from 0.2 to 0.8. Results were expressed as μmol TE/g DW using the same formula as the ABTS assay.

All antioxidant assays included generation of dispersion graphs to illustrate the relationship between concentration and absorbance for each sample.

### 2.5. Assesment of In Vitro Antiplatelet and Anti-Inflammatory Properties of the Supplements with Light Transmittance Aggregometry

The antiplatelet and anti-inflammatory activities of the supplements were evaluated using human platelet-rich plasma (hPRP) obtained from healthy donors. The inhibitory effect of the supplements on platelet aggregation was assessed following stimulation with platelet-activating factor (PAF) and adenosine diphosphate (ADP) agonists in the presence of the test compounds, as described in [48]. Results were expressed as mean IC_50_ values ± standard deviation, representing the concentration of bioactive compound required to achieve 50% inhibition of platelet aggregation. These values were quantified based on the mass of active compounds and normalized per gram of vitamin C and flavonoid content in the vitamin C and bioflavonoid supplement and μΜ for vitamin C-only supplement. Each sample was tested multiple times using blood samples from different healthy donors to ensure reproducibility and reliability.

### 2.6. Assessment of In Vivo Antiplatelet and Anti-Inflammatory Properties of the Supplements with Light Transmittance Aggregometry

#### 2.6.1. Study Design—Participants

A total number of N = 20 healthy volunteers were enrolled in the clinical trial and randomly separated into two different groups (randomized two arms study). Healthy subjects were recruited based on the following inclusion criteria: absence of ongoing medication, overall health status, no intake of similar supplements or vitamin supplements within the previous month, comparable age range, and similar lifestyle habits. Thus, prior to participating in the study and before the administration of any type of supplement, all participants were fully informed about the study and signed their consent form, while they also completed a detailed questionnaire collecting personal information, family history of serious diseases, recent surgeries or medications, supplement use, lifestyle habits (including smoking and alcohol consumption), and dietary intake of vitamin-rich foods such as animal food (meat, dairy), fruits, and vegetables. Subsequently, baseline anthropometric measurements including height, weight, and normal body mass index (BMI) were also recorded. Subsequently, two participants were excluded from each group for not meeting the inclusion criteria or due to infections at the time period that the trial took place.

The first group (VC) consisted of N = 7 subjects that were orally administered a 1 g vitamin C-containing supplement for 28 days (four weeks), while the second group (VCF) consisted of N = 8 subjects that were orally administered a 1 g vitamin C supplement that was enriched with 50 mg of citrus and rose flavonoids for 28 days (four weeks). Supplement administration commenced following the initial blood draw, designated as the baseline, and continued daily for 28 days, with participants ingesting one tablet of the assigned supplement each day.

#### 2.6.2. Study Design—Blood Analysis

Blood samples were collected from all participants at baseline (day 0), prior to supplementation (t = 0), and again after 28 days of daily supplementation of VC or VCF. Platelet aggregation was evaluated using three agonists: PAF, ADP, and thrombin, according to Tsoupras et al. [48]. Immediately after collection, blood samples collected in citrate containing monovette tubes were centrifuged at 194× *g* for 18 min at 24 °C to isolate platelet-rich plasma (PRP). The remaining blood was subsequently centrifuged at 1465× *g* for 20 min at 24 °C to obtain platelet-poor plasma (PPP). PRP and PPP were separated into distinct tubes for further analysis. For aggregometry assays, 250 μL of PRP containing a magnetic stir bar and 500 μL of PPP without stir bar were transferred to each aggregometer cuvette and placed at the appropriate positions at the aggregometer. Platelet aggregation was quantified by determining the mean EC_50_ values, representing the concentration of each agonist required to induce 50% platelet aggregation. These values were normalized per gram of total content, vitamin C, and flavonoid content in relation to the vitamin C and flavonoid supplement. The change in EC_50_ values after 28 days of supplementation provided an indication of the supplement’s modulatory effect on platelet aggregation.

### 2.7. ATR-FTIR Analysis

In order to obtain the spectra of the supplements themselves and in comparison with phenolic standards, we utilized the ATR-FTIR spectrophotometry technique using a Perkin Elmer Frontier ATR/FT-NIR/MIR spectrometer (PerkinElmer, Inc., Shelton, CT, USA) in a wavenumber range of 4000–600 cm^−1^. The supplements for this technique were not dissolved and prepared like the rest assay. In contrast, we just grinded the supplement using a porcelain mortar and placed a small amount in the crystal of the device. Then, after contact with the sample, we set the Force Gauge at approximately 80%.

### 2.8. Wavelength Scan

The UV-Visible wavelength scan of the vitamin C and flavonoid supplement was performed using ascorbic acid as the reference standard and deionized water for baseline calibration. The supplement sample was diluted at a ratio of 1:1000 to obtain absorbance readings within the range of 190–3000 nm.

### 2.9. Statistical Analysis

For the in vitro assessment, based on descriptive statistics, data are expressed as means (±SD). The Kolmogorov–Smirnov test was applied to assess the normality of distribution, which informed the selection of appropriate statistical analyses. A one-way analysis of variance (ANOVA) was employed to compare the normal distributed IC_50_ values related to agonist-induced platelet aggregation in human samples. For any other comparisons, one-way ANOVA was also applied to compare means of normal distributed parameters, while for non-parametric distributions of data, means were compared by using the Kruskal–Wallis non-parametric test for independent samples. All these analyses were performed using IBM SPSS Statistics for Windows, Version 29.0 (IBM Corp., Armonk, NY, USA).

For the in vivo study, the continuous variable (EC_50_) was summarized using mean (standard deviation-SD). Given the experimental design, which involved two treatment groups (VC and VCF) with repeated measurements per subject at two time points (Day 0 and Day 28), Linear Mixed Effects (LME) models were used, including a random intercept for each subject to account for the intra-subject correlation due to repeated measures. To examine the effects of the experimental conditions, Time and Group were included as fixed effects in the model, along with their interaction term (Time × Group) to assess whether differences in EC_50_ over time were observed between groups. The full model, which included the interaction term, was then compared to a reduced model containing only the main effects of Time and Group via the Likelihood Ratio Test (LRT). If the interaction term was not statistically significant, the more parsimonious model including only the main effects was retained as the final model. Furthermore, the final model was examined for potential effects of covariates (Gender, Μedication/Supplements, Smoking, Vegetables, Fruits, Beverages, and Physical Activity) using a sequential forward selection approach. At each step of the approach, the covariate that produced the greatest reduction in the Akaike Information Criterion (AIC) along with a statistically significant improvement in model fit based on LRT was added to the model. This process was repeated, iteratively, until no additional covariates met the inclusion criteria. Estimated marginal means were computed followed by post hoc comparisons using Tukey’s adjustment to control family-wise Type I error rate. Finally, model assumptions, i.e., normality of residuals and homoscedasticity, were checked via appropriate diagnostics, e.g., Q-Q plots and residuals against fitted value plots. All statistical analyses were performed using R (R Core Team, 2025 [49] through lme4, lmerTest and emmeans packages). All statistical tests were non-directional, and a difference was considered significant if the *p* value was less than 0.05 (a = 0.05).

## 3. Results

### 3.1. Qualitative and Quantitative Detection of Flavonoids in the VCF Supplement

Several commercially purchased supplements claim that they contain specific bioactives, but this is not always true. Since we purchased the VCF supplement from a local pharmacy, before administering it to the healthy subjects, we initially performed qualitative analysis using UV-Vis and ART-FTIR to detect the phenolic compounds in the flavonoids group within this supplement and we also quantified these phenolics according to the Folin–Ciocalteu methodology. Both qualitative and quantitative assessment revealed the presence of 30–50 mg of flavonoids in this supplement. We outline this more specifically in the following:

#### 3.1.1. Results of the Spectrophotometric Wavelength Scan

**Figure 1:**
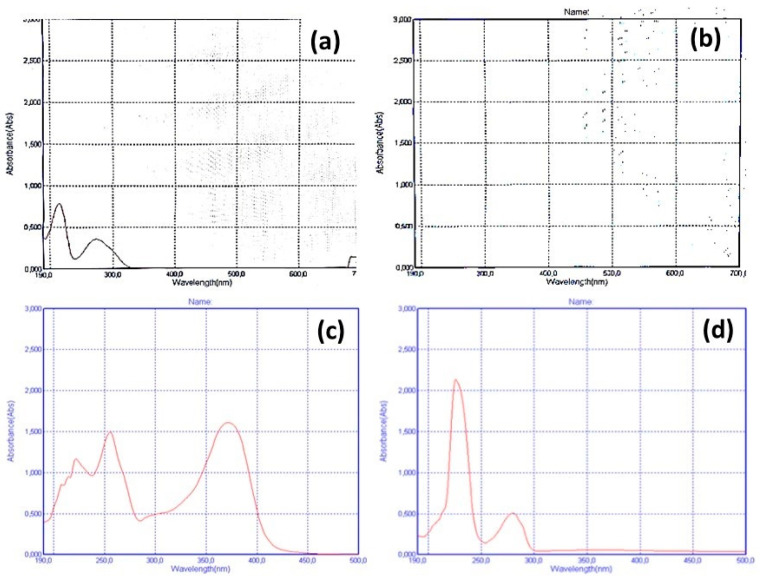
UV-Vis spectrum of vitamin C and flavonoids of the VCF supplement **(a**) and that of standards ascorbic acid (**b**), quercetin (**c**), and catechin, (**d**) (in this figure commas in all numbers depict decimal points according to the Greek alpha-metric system that the spectrophotometer has been set).

As shown in Figure 1, wavelength scan analysis of the VCF supplement showed that it exhibits different peaks at the UVA and UVB regions, which are a result of the absorption of the UVA and UVB radiation from the flavonoids content of this supplement; this is in comparison to the wavelength scan analysis of standard flavonoids that showed similar results, as well as the comparison of the ascorbic acid standard that showed no absorption at the UV regions. Thus, these results indicate the presence of flavonoid phenolics in the VCF supplement. Nevertheless, to ascertain more information, we further analyzed the supplement with ATR-FTIR analysis.

#### 3.1.2. Results of the ATR-FTIR Analysis

Fourier-transform infrared (FTIR) spectroscopy was utilized to characterize the structural profile of the VC supplement in comparison to a reference spectrum of pure ascorbic acid, with the aim of assessing compositional purity; the same process was performed for VCF in comparison to phenolic standards (Supplementary Figures S1–S8 in the Supplementary File) in order to ascertain their presence in the VCF supplement.

**Figure 2:**
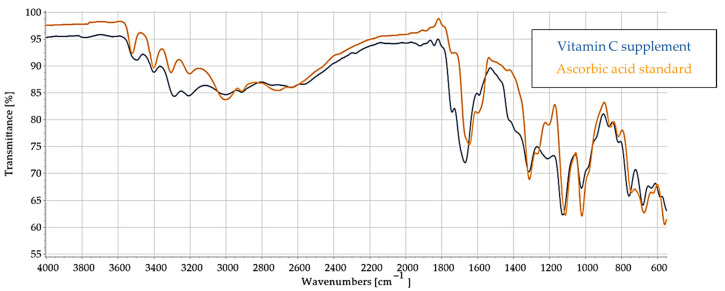
Vitamin C supplement and ascorbic acid standard comparison.

In the VC supplement spectrum (Figure 2), a broad and strong absorption band in the region of 3500–3200 cm^−1^ was observed, corresponding to O–H stretching vibrations, which are indicative of the multiple hydroxyl groups and are characteristic of ascorbic acid. The presence of aliphatic C–H stretching was confirmed by peaks in the 3000–2850 cm^−1^ range. A prominent, sharp absorption peak around 1750 cm^−1^ was attributed to the C=O stretching vibration of the lactone ring, a distinct structural feature of ascorbic acid. Additional peaks at 1450–1350 cm^−1^ were consistent with C–H bending vibrations, while the region from 1300 to 1000 cm^−1^ exhibited multiple absorption bands corresponding to C–O stretching and O–H bending, confirming the presence of alcohol and ether functionalities. The fingerprint region (950–700 cm^−1^) showed complex bending vibrations typical of the ascorbic acid molecule. The spectra of the pure ascorbic acid standard (Figure 2) also revealed all the characteristic peaks of ascorbic. However, notable differences between the two spectra in peak intensity and shape were evident.

**Figure 3:**
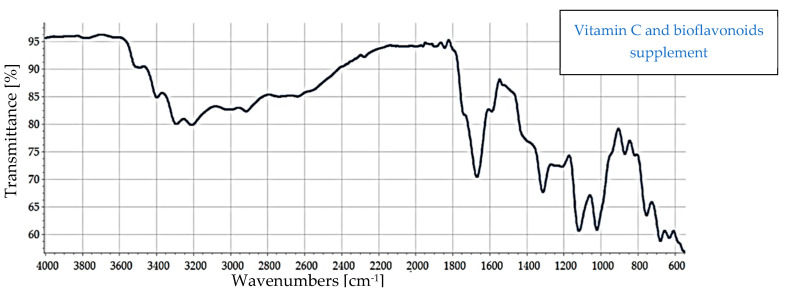
Vitamin C and bioflavonoids supplement FTIR spectrum, with peaks resembling the characteristic groups in the compounds of the supplement.

The FTIR spectrum of the dietary supplement enriched with vitamin C, citrus, and rose bioflavonoids was analyzed to identify the presence of phenolic compounds and assess functional group characteristics, with comparisons made against reference spectra of known phenolics (Figure 3). The obtained spectrum offers valuable insight into the molecular composition and potential interactions among the supplement’s bioactive constituents.

A broad absorption band observed in the region of 3300–3400 cm^−1^ is indicative of O–H stretching vibrations and characteristic of hydroxyl groups. These functional groups are abundant in both vitamin C and polyphenolic compounds such as flavonoids, and the breadth of the band suggests extensive hydrogen bonding, potentially due to intermolecular interactions between vitamin C and flavonoid hydroxyl groups. In the 2900–2850 cm^−1^ region, aliphatic C–H stretching bands were evident, which are common in the hydrocarbon backbones of organic molecules, including flavonoids.

A pronounced carbonyl (C=O) stretching band was present between 1680 and 1750 cm^−1^, typically associated with ester or lactone groups. This feature is consistent with structural motifs found in both vitamin C (notably the lactone ring) and certain flavonoids or their glycosylated derivatives. Additionally, the 1600–1650 cm^−1^ region exhibited peaks corresponding to C=C stretching in aromatic systems, suggesting the presence of aromatic rings, a defining characteristic of many flavonoids such as quercetin, hesperidin, and catechin, which are likely constituents of the supplement.

Further peaks in the 1200–1000 cm^−1^ region were assigned to C–O–C stretching vibrations, indicative of ether or glycosidic linkages. These may arise from the presence of glycosylated flavonoids, as well as the ether functionalities within the vitamin C molecule itself. The presence of these characteristic peaks supports the conclusion that the supplement contains a complex matrix of bioactive phenolic compounds, with notable representation of flavonoid glycosides, in addition to vitamin C and associated phytoconstituents from rose hips.

To further investigate the compositional profile of the supplement, the FTIR spectrum of the vitamin C–flavonoid supplement (Figure 3) was compared with that of pure ascorbic acid (Supplementary Figure S9 at the Supplementary File). Both spectra share several characteristic absorption bands, confirming the presence of ascorbic acid as a key component of the supplement. Specifically, a broad O–H stretching band observed in the 3200–3600 cm^−1^ range appears in both spectra, indicative of extensive hydrogen bonding among hydroxyl groups—a signature feature of ascorbic acid. Additionally, both spectra exhibit a prominent C=O stretching band between 1650 and 1750 cm^−1^, associated with the lactone carbonyl group, further substantiating the presence of ascorbic acid.

Moreover, absorption bands located in the fingerprint region (1500–600 cm^−1^) are evident in both spectra. These peaks, corresponding to C–O stretching and C–H bending vibrations, are common in both vitamin C and flavonoid structures, supporting the presence of shared structural motifs.

However, the supplement spectrum (Figure 3) demonstrates additional peaks not found in the pure ascorbic acid standard. These extra bands are likely attributable to the presence of flavonoids and other phenolic compounds, such as those derived from citrus bioflavonoids and rose hips. Furthermore, the supplement spectrum exhibits broader and less defined peaks, particularly in the O–H and fingerprint regions. This broadening may result from intermolecular interactions—such as hydrogen bonding—between ascorbic acid and co-existing flavonoids, as well as from matrix complexity and physical heterogeneity (e.g., amorphous content, excipients, or hygroscopic behavior).

These spectral differences provide strong evidence that the supplement is not a simple ascorbic acid formulation, but a multicomponent matrix with distinct chemical interactions and contributions from various phenolic constituents. The spectral profile reinforces the supplement’s enriched composition and the potential synergistic interactions among its bioactive components.

Thus, the supplement spectrum (Figure 3) confirms the presence of ascorbic acid due to strong similarities with the standard spectrum. All the additional peaks indicate the presence of other compounds, specifically flavonoids from citrus and rose. Since we do not know the exact composition of the flavonoids in the supplement, we also made a comparison with the tested samples of the phenolic compounds in our research in order to obtain any common peaks to clarify the composition.

As illustrated in Table 2, the FTIR spectrum of the vitamin C-enriched supplement reveals multiple characteristic absorption bands that are consistent with the structural features of flavonoids and phenolic compounds such as quercetin and catechin (Supplementary Figures S4 and S5 at the Supplementary File). A broad and intense O–H stretching band centered around 3300 cm^−1^ is observed, indicative of hydroxyl groups commonly found in both vitamin C and polyphenols. Additionally, the presence of a C=C aromatic stretching band near 1600 cm^−1^ supports the presence of aromatic rings, a hallmark of flavonoid structures. Peaks corresponding to carbonyl (C=O) and ether (C–O–C) groups, which are key functional moieties in many flavonoids, are also detected and align with those found in reference compounds.

These spectral correlations confirm the presence of bioactive flavonoids in the supplement formulation. Moreover, variations in peak intensity and shape between the supplement and individual standards may reflect intermolecular interactions between vitamin C and flavonoids. Such interactions could influence the bioavailability and stability of the active compounds, potentially enhancing the supplement’s overall efficacy.

Nonetheless, while FTIR provides qualitative insights into functional group composition, further analytical investigation—such as HPLC-DAD or LC-MS/MS—would be required to accurately quantify individual flavonoids and elucidate their precise interactions within the matrix.

#### 3.1.3. Quantification of the Total Phenolic Content of VCF

The total phenolic content of the vitamin C and flavonoid-enriched supplement (VCF) is expressed as milligrams of gallic acid equivalents (mg GAE) per gram of sample and ranged from 32 to 38 mg GAE/g. These findings indicate a substantial presence of phenolic compounds, likely attributable to the supplement’s enrichment with bioactive flavonoids. The narrow variability in phenolic content across samples suggests a high degree of formulation consistency and reproducibility. Flavonoids, a major subclass of phenolic compounds included in the supplement, are well-documented for their potent antioxidant properties and have been extensively associated with anti-inflammatory, cardioprotective, and anticancer effects. Furthermore, the co-administration of vitamin C with flavonoids may enhance the stability and bioavailability of phenolic compounds, potentially increasing the overall efficacy of the formulation.

### 3.2. Antioxidant Activity

The antioxidant capacity of standard phenolic compounds (simple phenolics, flavonoids, and polyphenols) of the supplement containing vitamin C (VC) and that of the supplement containing vitamin C and flavonoids (VCF) were evaluated using three complementary analytical assays, FRAP, DPPH, and ABTS, each targeting distinct chemical mechanisms. This multimodal approach enabled a comprehensive assessment of the compounds’ antioxidant potential. Collective results are shown in Table 3.

Distinct antioxidant profiles were observed across the phenolic compounds tested, varying by assay methodology. In the FRAP assay, which assesses the reducing power of antioxidants, quercetin exhibited the highest activity, indicating a strong capacity to reduce the ferric-tripyridyltriazine complex. The low standard deviation relative to its mean suggests high stability and excellent measurement repeatability. Curcumin followed closely with comparably high reducing power and similarly low variability, underscoring its robustness as an antioxidant. Catechin demonstrated moderate activity with commendable consistency across replicates. Conversely, tannic acid and gallic acid exhibited weaker reducing abilities, though with low standard deviations, indicating stable but less potent antioxidant potential. Thymol and vanillin were the least effective in this assay and showed greater variability, implying poor stability. Tannin, while traditionally recognized for its antioxidant potential, showed both low FRAP activity and high measurement variability, suggesting sensitivity to external factors and reduced assay reliability.

In the ABTS assay, gallic acid emerged as the most potent compound, characterized by high activity and minimal standard deviation. Thymol also displayed satisfactory antioxidant performance with strong reproducibility. While quercetin retained high radical scavenging capacity, it was accompanied by a larger standard deviation, indicating greater variability and potential sensitivity to assay conditions. Curcumin showed moderate ABTS activity with acceptable stability. Tannin, despite strong radical scavenging ability, again demonstrated poor reproducibility, as indicated by its high standard deviation. Catechin, vanillin, and tannic acid had the lowest ABTS activities, suggesting limited effectiveness in neutralizing the ABTS radical cation under the assay conditions.

In the DPPH assay, which favors hydrophobic and stable radical-scavenging compounds, the highest activity was recorded for tannin, tannic acid, gallic acid, and catechin. These compounds not only achieved the highest mean values but also exhibited low standard deviations, highlighting both their potent antiradical capabilities and excellent stability. Thymol demonstrated moderate activity with reasonable consistency. Vanillin displayed weak radical-scavenging potential and is thus not well-suited for this assay. Notably, quercetin, despite moderate activity, exhibited high variability, while curcumin showed both low antioxidant activity and high standard deviation, suggesting significant instability in this radical-based method.

These findings collectively underscore the compound-specific and assay-dependent nature of antioxidant behavior, with certain phenolics (e.g., quercetin, gallic acid) excelling in specific redox environments, while others (e.g., tannin, curcumin) display variable performance depending on assay chemistry and molecular stability. Nonetheless, as the results obtained from the FRAP assay are closer to the in vivo conditions of Fe in plasma, this seems to be the best criterion to categorize these phenolic bioactives assessed for their antioxidant capacity, with flavonoids like quercetin being the most representative antioxidant phenolics.

The evaluation of vitamin C’s antioxidant capabilities was confirmed by the values obtained from the previously mentioned complementary assays that were used, as demonstrated in the corresponding section of Table 3. Vitamin C from the VC supplement, which contains solely vitamin C, exhibited moderately high antioxidant activity in the FRAP assay, indicating its ability to reduce ferric (Fe^3+^) to ferrous (Fe^2+^) ions, a measure of its reducing power. In contrast, the DPPH assay, with limitations towards hydrophilic antioxidants, recorded comparatively low antioxidant activity. Finally, the ABTS assay is reactive toward both hydrophilic and lipophilic antioxidants, thereby providing a more inclusive and accurate representation of total radical-scavenging capacity, thus revealing a markedly higher antioxidant response.

To statistically compare the antioxidant capacities measured by each assay, the Kruskal–Wallis test for k independent samples was applied. The analysis yielded a statistically significant difference in antioxidant activity among the methods (*p* < 0.001), with mean rank values as follows: ABTS (16.00), FRAP (9.50), and DPPH (3.50). These findings demonstrate that the ABTS assay captured the highest antioxidant capacity, followed by FRAP, with DPPH measuring the lowest, confirming the antioxidant component of vitamin C but with a variation in behavior in each assay.

The antioxidant capacity of the tested solutions, comprising the vitamin C and bioflavonoid-enriched supplement, was assessed using three complementary assays: ABTS, DPPH, and FRAP. The results, as summarized in Table 3**,** are expressed per gram of vitamin C, per gram of flavonoid extract, and for the total supplement. Across all assays, the flavonoid fraction consistently demonstrated superior antioxidant activity compared to both vitamin C alone and the complete supplement formulation.

DPPH assay revealed the highest radical scavenging activity for flavonoids, a trend that was similarly observed in the ABTS and FRAP assays. Although the total sample exhibited marginally higher activity in the DPPH assay compared to vitamin C alone, its antioxidant capacity was lower than both the flavonoid and vitamin C fractions in the ABTS and FRAP assays, indicating that flavonoids are more stable and reactive under the assay conditions used, likely due to their polyphenolic structure and broader interaction spectrum with oxidative species. In contrast, vitamin C, being less stable and more assay-sensitive, showed reduced performance, particularly in lipophilic environments such as that represented by the DPPH assay.

To evaluate statistical significance, a non-parametric Kruskal–Wallis test was applied. The analysis revealed statistically significant differences, both between assays and among the supplement fractions within each assay (*p* < 0.05), underscoring the distinct antioxidant profiles of the individual components. Collectively, these results reinforce the potent antioxidant capacity of flavonoids and suggest that their efficacy may not be enhanced when co-administered with vitamin C under the tested conditions.

### 3.3. In Vitro Anti-Inflammatory and Antithrombotic Activity

The standard phenolic compounds used in the study were evaluated for their anti-inflammatory activity through the IC_50_ (inhibitory concentration 50%) value against platelet accumulation induced by the platelet activating factor PAF (Platelet Activating the Factor Activation). The descriptive statistics of each substance for the specific biological pathway are presented in Table 4, where values refer to IC_50_ in µM.

The analysis of IC_50_ values for platelet-activating factor (PAF) inhibition revealed that the flavonoids catechin and quercetin exhibited strong inhibitory effects, with IC_50_ values ranging from 137.81 to 689.04 µM and from 132.35 to 782.07 µM, respectively. Significant inhibition was also observed for the polyphenolic compound tannic acid, which showed an IC_50_ range from 551.08 to 646.61 µM. Notably, the dimeric phenol curcumin demonstrated the strongest inhibitory effect, with an IC_50_ value range from 176.45 to 268.27 µM.

In contrast, simpler phenolic biomolecules such as thymol (ranging from 961.55 to 9319.66 μΜ), gallic acid (ranging from 6298.08 to 16981.48 μΜ), and vanillin (ranging from 1627.47 to 7886.95 μΜ) displayed much higher mean IC_50_ values, indicating lower biological activity against PAF-mediated platelet aggregation. Their inhibitory effects were limited and accompanied by considerable variability, especially for gallic acid and thymol, as reflected by larger standard deviations. The second polyphenol evaluated, tannin, ranged from 321.75 to 1266.07 μΜ, showing moderate inhibitory activity against PAF.

Furthermore, the standard phenolic compounds were also assessed for their antithrombotic potential by determining IC_50_ values against platelet aggregation induced by thrombin, a key pro-coagulant factor in the coagulation cascade.

The analysis of IC_50_ values for thrombin-induced platelet aggregation inhibition demonstrated that the flavonoid compounds catechin and quercetin exhibited highly significant inhibitory effects, with an IC_50_ value range from 126.32 to 175.39 µM and from 77.85 to 168.44 µM, respectively. Similarly, curcumin showed remarkable inhibition with IC_50_ values ranging from 58.47 to 304.03 µM. The low standard deviations observed for these compounds indicate good experimental repeatability and stability. Among them, quercetin appears to be the most potent inhibitor of thrombin-induced platelet aggregation based on its lowest mean IC_50_ value.

In contrast, simpler flavonoid molecules, like vanillin (values ranging from 1445.94 to 4819.81 μΜ), thymol (values ranging from 4184.34 to 12553.02 μΜ), and gallic acid (values ranging from 2351.28 to 3233.01 μΜ) displayed considerably higher IC_50_ values, reflecting weak inhibitory activity against thrombin, with thymol showing the weakest effect (IC_50_ = 9200.53 µM). The polyphenolic compounds tannin and tannic acid exhibited moderate inhibitory activity, resulting in partial inhibition of thrombin-induced aggregation.

The model phenolic compounds were evaluated for their antiplatelet activity against platelet aggregation induced by ADP, a common platelet agonist. The analysis of IC_50_ values for inhibition of platelet aggregation induced by ADP revealed that the phenolic model substances exhibited inhibitory patterns similar to those observed against thrombin-induced aggregation. Notably, curcumin demonstrated the strongest inhibitory effect, with an IC_50_ range from 73.68 to 180.97 μM, followed closely by quercetin, which showed a strong effect with IC_50_ values ranging from 86.03 to 205.88 μM.

In contrast, the simple phenolic molecules gallic acid, thymol, and vanillin exhibited very weak activity in this assay, as evidenced by their high mean IC_50_ values. Among them, thymol (values ranging from 3643.78 to 10,983.89 μΜ) showed the weakest inhibitory effect, with an IC_50_ of 7615.95 μM, indicating minimal efficacy.

The polyphenolic tannic acid demonstrated a relatively strong inhibition, with an IC_50_ range from 42.55 to 679.26 μM, whereas catechin (values ranging from 1309.17 to 6545.86 μΜ) and tannin (values ranging from 203.07 to 775.92 μΜ) showed moderate inhibitory effects with intermediate IC_50_ values.

In summary, across the assays targeting PAF, ADP, and thrombin-induced platelet aggregation, quercetin and curcumin consistently exhibited the strongest inhibitory activities. Catechin also showed potent inhibition against PAF- and thrombin-induced aggregation, while tannic acid was notably effective against ADP-induced aggregation. Simple phenolic molecules, however, displayed weak or negligible antiplatelet activity across all three assays.

To assess the in vitro anti-inflammatory and antiplatelet properties of vitamin C, the half-maximal inhibitory concentration (IC_50_) was determined for platelet aggregation induced by two key agonists: Platelet-Activating Factor (PAF) and Adenosine Diphosphate (ADP), and the results are presented in Table 4, in both μM and μg. The IC_50_ value indicates the concentration of vitamin C required to inhibit 50% of platelet aggregation, with lower IC_50_ values reflecting stronger inhibitory efficacy.

Vitamin C exhibited a range of 999.32–2465.42 in μM and 44–108.552 in μg, a notably lower IC_50_ in response to PAF-induced platelet aggregation. Conversely, a comparatively weaker response was found by the higher IC_50_ value observed for ADP-induced aggregation, with values ranging from 929.12 to 4826.26 in μM and 40.90 to 212.5 in μg.

With respect to the IC_50_ values between PAF and ADP, the results indicated no significant difference between groups (F(1, 12)=1.260, p=0.284), suggesting that variability within the data prevented the detection of a statistically robust effect at the conventional α-level of 0.05. Effect size estimates further contextualize this finding. The Eta-squared ($\eta^{2}$) value was 0.095, indicating a small effect size, while Epsilon-squared ($\varepsilon^{2}$) and Omega-squared ($\omega^{2}$) values were both 0.020 and 0.018, respectively, underscoring the modest and statistically uncertain influence of agonist type on vitamin C’s antiplatelet activity. Moreover, some of the confidence intervals for these estimates were zero, reinforcing the conclusion that the observed differences could be attributed to random variation rather than a consistent biological effect.

Multiple comparisons with ANOVA and LSD post hoc tests revealed differences in the impact of each compound on IC_50_ values, expressed per mg of vitamin C, flavonoids, or total bioactive content, in samples stimulated with either PAF or ADP. The results, summarized in Table 4, revealed a statistically significant effect of group on IC_50_ values ($F\left(5,57\right)=45.59,\;p<0.001$), indicating that mean inhibition concentrations differed markedly depending on both the agonist and normalization method. Supporting this finding, the effect size was large, with an Eta-squared ($\eta^{2}$) of 0.800, suggesting that 80% of the variance in IC_50_ values was explained by the grouping factor. Corresponding epsilon-squared ($\varepsilon^{2}=0.782$) and omega-squared ($\omega^{2}=0.780$) values further confirmed the substantial influence of agonist type and normalization on the inhibitory potency of the supplement. According to the group effect, PAF led into a range of inhibition of 7.06 to 53.33 in the vitamin C group, while the flavonoids group had a range from 0.35 to 2.67 μg. The ADP groups also had a wide range of values, with the vitamin C group ranging from 225 to 900 μg while the flavonoid group was quite lower, with a significant difference from 11.25 to 45.00 μg.

Post hoc pairwise comparisons demonstrated multiple statistically significant differences between groups (p<0.05). Notably, all PAF-based samples (total range 7.41 to 56.007 μg) exhibited IC_50_ values significantly different from ADP-based samples (total range 236.25 to 900.00 μg), (p<0.001), underscoring the strong impact of agonist choice on inhibition outcomes. This suggests that there were distinct mechanisms in platelet activation pathways or differential modulation instigated by the bioactive compounds tested. Within the PAF group, bioflavonoids had statistically significant lower IC_50_ values, and thus a much stronger anti-PAF action, than vitamin C in the supplement. It is important to note that the IC_50_ values that are calculated for this supplement seem to be some of the lowest in the bibliography. According to the results in Table 4, bioflavonoids seem to have the lowest IC_50_ values when compared to either phenolic or vitamin C-only supplement. These results suggest that bioflavonoids have strong activity, but also sensitivity, in the inhibition of the inflammatory PAF-pathway, while the presence of vitamin C also enhances this activity and overall efficacy against this inflammatory agent.

In summary, flavonoid inclusion in the supplement improves total antiplatelet activity, supporting the synergistic activity of flavonoids and vitamin C and making their use a viable option in the treatment of cardiovascular diseases or in prevention strategies as natural agents-competitors, especially in pathways involving PAF or ADP.

It is worth noting that the IC_50_ values of the flavonoids-enriched supplement in the PAF and ADP pathways have statistically significant differences, with PAF IC_50_ values being much lower and showing a specialization in PAF that, as mentioned, is enhanced by synergistic mechanisms between the components. These in vitro results are very important, since PAF inhibition, which is caused by the synergy between amphiphilic bioactives like flavonoids and vitamin C, as observed in this study, are a much bigger class than is made clear in the bibliography. This discovery can promote strategies against inflammation, cardiovascular diseases, and cancer, in order to enhance healthcare treatments. Thus, in order to validate these results, a clinical trial was conducted.

### 3.4. In Vivo Anti-Inflammatory and Antithrombotic Health-Promoting Effects of the VC and VCF Supplements Administered for Four Weeks in Healthy Subjects

The overall effect of the VC and VCF supplements administered for four weeks in healthy subjects against thrombo-inflammatory platelet reactivity induced by PAF and classic thrombotic platelet reactivity induced by ADP or thrombin are shown in Table 5. Results are expressed as EC_50_ values (effective concentration that induces half maximum activation of platelets through a specific thrombo-inflammatory pathway/mediator) before and after 28 days of these administrations, as per [48]. The higher the EC_50_ value, the lower the platelet reactivity through this pathway, and thus the greater the anti-inflammatory (anti-PAF)/antiplatelet (anti-ADP)/antithrombotic (antithrombin) health-promoting properties of the compound, the presence of which increased the EC_50_ value of platelet reactivity induced by the specific pathway/mediator affected (Table 5).

**Figure 4:**
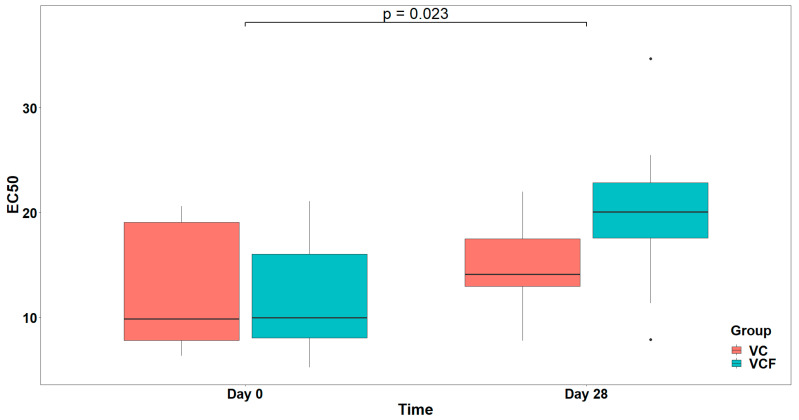
The anti-inflammatory (anti-PAF) health-promoting properties of the VC and VCF supplements prior to and after 28 days of administration. Results are expressed as EC_50_ values (nM). Statistically significant increase in EC50 after 1 month of this administration (main effect of Time on EC50) with *p* < 0.05.

**Figure 5:**
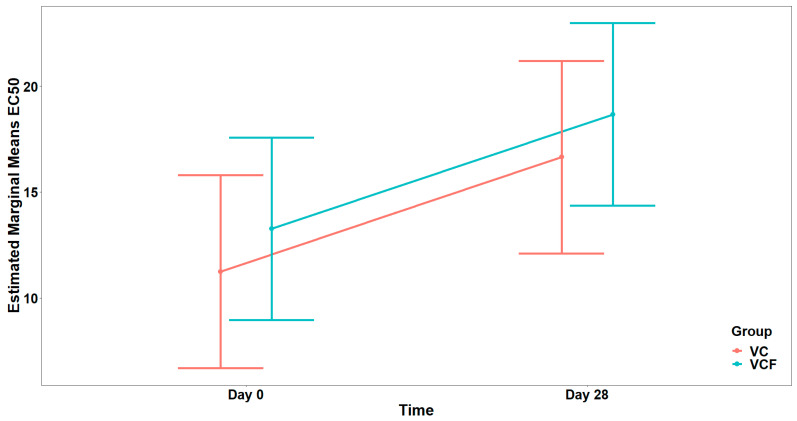
Estimated marginal means of EC_50_ for PAF (nM) by Group and Time with 95% confidence intervals.

Regarding PAF, visual inspection (Figure 4) of EC_50_ distributions suggested that participants in the VCF group exhibited, generally, higher EC_50_ at Day 28 compared to those in the VC group. However, comparison between the model with interaction term and the reduced model (main effect only) did not reveal a statistically significant interaction effect ($\chi^{2}\left(1\right)=2.608,\;p=0.106$). Therefore, the reduced model including only the main effects was retained as the final model, whereas none of the examined covariates (Gender, Medication/Supplementation, Smoking, and weekly frequency of consuming vegetables/fruits/beverages, and of physical activity/inactivity) presented a statistically significant improvement over the final model after applying the sequential forward selection process. The fitted model indicated a statistically significant main effect caused by Time on EC_50_ ($F\left(1,14\right)=6.553,\;p=0.023$), but no significant main effect caused by Group ($F\left(1,13\right)=0.597,\;p=0.454$) (Figure 5). The statistically significant main effect of Time in the rise in the EC_50_ values, against PAF, shows a lower activation of platelets from this agonist after 28 days of supplement contamination.

**Figure 6:**
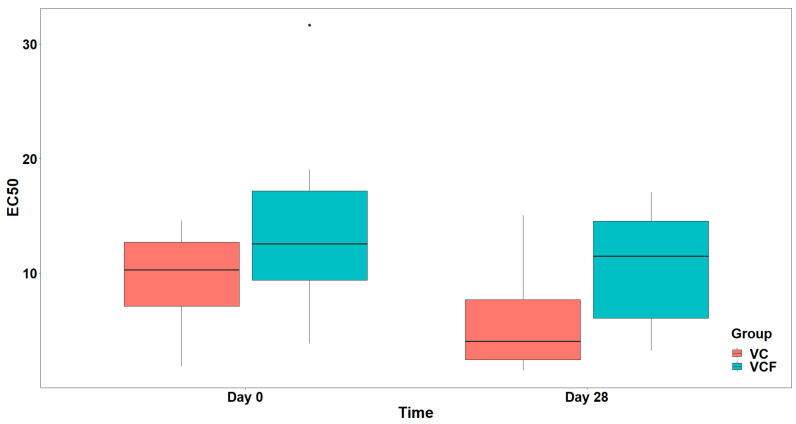
The antiplatelet (anti-ADP) health-promoting properties of the VC and VCF supplements prior to and after 28 days of administration. Results are expressed as EC50 values (μM).

**Figure 7:**
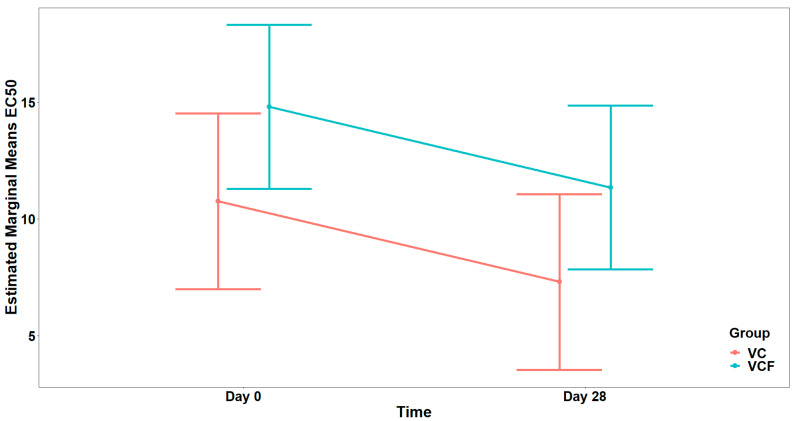
Estimated marginal means of EC_50_ for ADP (μM) by Group and Time with 95% confidence intervals.

For the agonist ADP (Figure 6), comparison between the full model (including the interaction term) and the model containing only the main effects did not show a statistically significant interaction effect ($\chi^{2}\left(1\right)=0.007,\;p=0.934$). In the final model, there was a marginally significant main effect from the Group ($F\left(1,26\right)=4.135,\;p=0.052$), while the main effect from Time was not statistically significant ($F\left(1,26\right)=3.059,\;p=0.092$), after adjusting for vegetable consumption ($F\left(1,26\right)=9.353,\;p=0.005$) (Figure 7).

**Figure 8:**
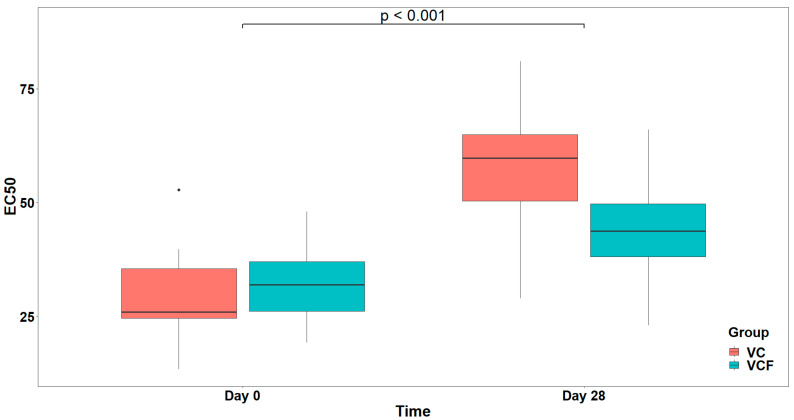
The antithrombotic (antithrombin) health-promoting properties of the VC and VCF supplements prior to and after 28 days of administration. Results are expressed as EC_50_ values for TRAP (thrombin receptor agonist peptide) (μM). A statistically significant increase in EC_50_ after 1 month of this administration (main effect of Time on EC50) is seen with p<0.001.

**Figure 9:**
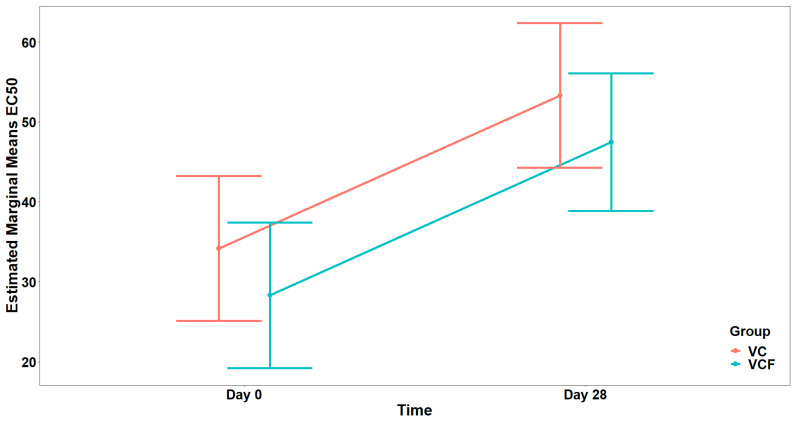
Estimated marginal means of EC_50_ for thrombin by Group and Time with 95% confidence intervals.

Finally, as far as the THROMBIN agonist (Figure 8) is concerned, the analysis did not reveal a statistically significant interaction between Time and Group, ($\chi^{2}\left(1\right)=2.642,\;p=0.104$). Consequently, the interaction term was not included in the final model. Τhe final model indicated a statistically significant main effect of Time on EC_50_ ($F\left(1,29\right)=19.805,\;p<0.001$) but no significant main effect of Group ($F\left(1,29\right)=2.546,\;p=0.121$) after adjusting for vegetable consumption ($F\left(1,29\right)=4.879,\;p=0.035$) (Figure 9).

Following the four-week intervention with the VC supplement, a modulation of platelet reactivity was observed, which suggests the presence of anti-inflammatory and antithrombotic effects. Specifically, the reduction in PAF-related platelet responsiveness was proved by the increase in the mean EC_50_ value for PAF-induced aggregation from 12.94 ± 6.33 to 14.96 ± 4.65, thus expressing a mild anti-inflammatory effect. In the case of thrombin-induced platelet aggregation, the increase in EC_50_ values was more pronounced, rising from 12.94 ± 6.33 to 14.96 ± 4.65, which highlights a considerable attenuation in thrombotic potential over time. In contrast, a decrease was recorded in ADP-induced platelet reactivity, where EC_50_ values dropped from 9.47 ± 4.59 to 5.84 ± 4.89. Despite these variable effects across pathways, the overall profile of the VC group shows a beneficial health-promoting activity following daily supplementation.

In the VCF group, improvements were more evident across inflammatory and thrombotic pathways, with EC_50_ values increasing in two out of three models after the 28-day intervention. A reduction in platelet responsiveness and strong anti-inflammatory activity was most significant in the PAF-induced model, where the EC50 value rose from 11.79 ± 5.51 at baseline to 20.14 ± 8.20 post-intervention. Similarly, thrombin-induced platelet aggregation was modestly reduced, with EC_50_ values increasing from 32.16 ± 9.67 to 44.10 ± 12.63, reflecting a beneficial antithrombotic effect. Unlike the VC group, the VCF group also exhibited a minor decrease in ADP-induced platelet reactivity, from 13.91 ± 8.95 to 10.62 ± 5.37, yet this change was less pronounced than in the VC group and did not negate the broader upward trend in EC50 values across the other pathways.

Comparative analysis of the two intervention groups reveals a stronger in vivo efficacy profile for the VCF supplement, particularly in the modulation of PAF-mediated platelet activation. While both VC and VCF groups demonstrated increases in EC_50_ values for PAF and thrombin, a more potent anti-inflammatory response was observed in the VCF group, which had a greater increase than that of the VC group. Notably, the decline in EC_50_ values for ADP was more pronounced in the VC group. However, statistical modeling indicated that while the PAF EC_50_ increase over time was significant, no significant group effect was found, while for ADP, only a marginal group effect was detected.

## 4. Discussion

In this section, we move to interpret and contextualize the key findings, exploring the potential roles of the active components of the supplements, the potential mechanisms of actions, and finally comparing our results with the existing literature to better understand the observed bioactivities and their implications.

The results of the FTIR analysis thoroughly revealed the purity, characteristics, and structural profiles of the supplements with the assistance of the standards. The spectral features observed in the VC supplement spectrum, such as the characteristic peaks of the multiple hydroxyl groups, the ketone group, and the typical bending vibrations of the fingerprint region, confirm the presence of ascorbic acid as the principal active component. When the spectrum of the supplement was compared to that of the pure ascorbic acid standard (Figure 2), all characteristic peaks of ascorbic acid were present in the supplement, confirming that ascorbic acid is its principal constituent. However, notable differences were identified in the comparison. The supplement displayed weaker and broader absorption bands, particularly in the O–H stretching region, compared to the sharp and intense peaks in the crystalline standard. These variations likely result from the presence of excipients in the supplement formulation, which reduces the relative concentration of ascorbic acid. Additional factors include increased hydrogen bonding, moisture content, and interactions between ascorbic acid and matrix components. Furthermore, the amorphous and heterogeneous nature of the supplement contrasts with the crystalline form of the reference compound, leading to increased light scattering and decreased spectral resolution. These spectral distinctions affirm that the supplement is a formulated mixture rather than a pure substance, with ascorbic acid as the primary active ingredient.

Further analysis of our results provided comprehensive insights into the phytochemical composition and biological potential of the vitamin C and bioflavonoid supplement. The total phenolic content analysis demonstrated a significant and reproducible level of phenolic compounds within the samples. Phenolic compounds, particularly flavonoids, are well-recognized for their potent antioxidant and health-promoting properties, and their presence in the supplement appears to enhance the efficacy of vitamin C. Specifically, the supplement exhibited strong radical scavenging activity, as confirmed by three distinct assays (DPPH, FRAP, and ABTS), with flavonoids consistently demonstrating superior antioxidant capacity across all methods.

The antioxidant capacity of phenolic biomolecules exhibited variability depending on the specific assay employed. Quercetin demonstrated a notably high reducing activity in the FRAP assay, yet its activity in the DPPH assay was comparatively low. Regarding the high antioxidant activity of quercetin in the FRAP test, Wenjun Pu et al. (2015) [50] also demonstrated in their study the strong activity of the compound in reducing the iron complex. Furthermore, Tian et al. (2020) [51] showed that the molecule is the strongest antioxidant (IC_50_ = 1840 μg/mL) compared to other flavonoids. In both of the above cases, the ability to destroy reactive oxygen species is demonstrated in the structure of the compound, specifically when considering the number of hydroxyl groups it has been compared to in the other flavonoid compounds evaluated.

Within the flavonoid class, catechin showed significant efficacy in the DPPH assay, thus, our results are in agreement with those of Pharm et al. (2024) [52], who isolated catechin from the bark extract of X. Moluccensis and presented the molecule as a powerful antioxidant with IC_50_ = 2.87 μg/mL, while Hou et al. (2022) [53], studying Msalais wine, rich in flavonoid molecules, showed strong activity in the DPPH test (0.918 µmol Trolox/L). The moderate activity of catechin in the FRAP assay appears to differ in the study of Ahmadi et al. (2020) [54], where catechin was compared to the compound luteolin, showing significantly stronger activity (689 µmol/L) in the FRAP assay by reducing the iron ion by a difference.

Additionally, gallic acid exhibited strong antioxidant activity in the DPPH and ABTS assays. Lee et al. (2015) [55] also present gallic acid as a powerful antioxidant molecule with an activity of over 90% (IC_50_ = 1.03 μg/mL). However, its capacity to reduce the iron complex in the FRAP assay was moderate.

Vanillin was the only phenolic compound that exhibited consistently low activity across all antioxidant assays. Our results in the DPPH assay are similar with Tai et al. (2011) [56], where this phenolic molecule showed no activity in the DPPH assay.

Thymol demonstrated notable activity in the ABTS assay, which is consistent with Mohamed Taibi et al. (2024) [57], where the phenolic molecule individually exhibited good antioxidant capacity but its performance in the FRAP and DPPH assays was moderate.

Curcumin, characterized as the simplest dimeric phenolic compound, showed remarkable activity in the FRAP assay but was inactive in the DPPH assay. Although curcumin is characterized as a molecule with strong antioxidant activity, in our experimental study it showed the lowest activity in the DPPH test. Asouri et al. (2013) [58] showed in their study that curcumin is a powerful antioxidant molecule compared to ascorbic acid, with a radical scavenging rate of 83% and an IC_50_ value of 53 μM (ascorbic acid IC_50_ = 83 μM). Curcumin was a compound with moderate activity in the present test, as no significant inhibition of ABTS cationic radicals was achieved. These results agree with the study by Eldiasty et al. (2024) [59], which also highlighted curcumin as a moderately potent antioxidant compound with a value of 670.20 ± 12.70 μmol Trolox eq/g at a concentration of 100 µg/mL of pure compound.

Both tannin and tannic acid displayed strong and comparable activity in the DPPH assay. The results from the evaluation of tannic acid activity agree with Jing et al. (2019) [60], where the molecule showed strong scavenging abilities in this test, over 90%, even in a small concentration range of 2–14 µg/mL. Similarly, the strong antioxidant activity of tannin agrees with Victoria Vorobyova et al. (2023) [61], who showed that the tannin extract of the Quebracho plant has a remarkable ability to destroy free radicals. However, their performances diverged in the ABTS assay, with tannin exhibiting substantial radical scavenging ability while tannic acid demonstrated relatively weak activity. The tannic acid molecule is characterized by strong antioxidant capacity against ABTS radicals according to Yingjun Jing et al. (2019) [60], which contradict our findings that the molecule exhibits low antioxidant activity. Furthermore, Chen et al. (2023) [62] present tannic acid as the strongest antioxidant for concentrations between 10 and 0.1563 mM and a scanning rate above 80%.

This outcome is consistent with the known hydrophilic nature of vitamin C, which limits its reactivity with the lipophilic DPPH radical, thus highlighting the limitations of the assay for evaluating water-soluble antioxidants. Conversely, the ABTS assay revealed a markedly higher antioxidant response. The ABTS radical cation is reactive toward both hydrophilic and lipophilic antioxidants, thereby providing a more inclusive and accurate representation of total radical-scavenging capacity. This pattern aligns with findings from citrus pulp analyses, where vitamin C content demonstrated a much stronger correlation with antioxidant capacity measured by ABTS (r^2^ = 0.91) than with DPPH (r^2^ = 0.85), reinforcing the superior sensitivity of the ABTS assay for detecting hydrophilic antioxidants like vitamin C [63]. The elevated antioxidant activity observed in the ABTS assay thus underscores the broad-spectrum efficacy of vitamin C across diverse reactive species, while also confirming that DPPH may significantly underreport the antioxidant strength of such compounds due to its solvent selectivity and radical nature. Similarly, in another study [64] vitamin C again showed limited antioxidant activity in the DPPH assay in comparison to the FRAP assay, which reliably reflected a higher antioxidant capacity. Although FRAP is often used to evaluate the electron-donating capacity of antioxidants, it can underestimate the activity of compounds like vitamin C if sample handling or extraction is suboptimal. In summary, the ABTS assay appears to be the most effective for assessing the antioxidant activity of vitamin C, due to its broader reactivity profile. The FRAP assay provides valuable insight into reducing potential but may underestimate antioxidant strength due to its narrower specificity. The DPPH assay, although commonly used, is less suited for evaluating hydrophilic antioxidants like vitamin C and may significantly underreport their activity.

Comparing the enriched supplement with bioflavonoids and the vitamin C supplement, it showed the most superior activity, leading us to find synergistic mechanisms. The antioxidant capacity of the tested solutions—comprising the vitamin C and bioflavonoid-enriched supplement—was assessed using three complementary assays: ABTS, DPPH, and FRAP. Across all assays, the flavonoid fraction consistently demonstrated superior antioxidant activity compared to both vitamin C alone and the complete supplement formulation. Notably, the DPPH assays revealed the highest radical scavenging activity for flavonoids, a trend that was similarly observed in the ABTS and FRAP assays. Although the total sample exhibited marginally higher activity in the DPPH assay compared to vitamin C alone, its antioxidant capacity was lower than both the flavonoid and vitamin C fractions in the ABTS and FRAPS assays. These findings suggest the absence of a synergistic or additive effect when vitamin C and flavonoids are combined within the same formulation. The data indicate that flavonoids are more stable and reactive under the assay conditions used, likely due to their polyphenolic structure and broader interaction spectrum with oxidative species. In contrast, vitamin C, being less stable and more assay-sensitive, showed reduced performance, particularly in lipophilic environments such as that represented by the DPPH assay. Specifically, even though flavonoids had an enhanced activity, vitamin C was not affected, acting as a protector in the oxidized environments in order to promote flavonoids activity.

In order to further investigate these results, we used ex vivo models, to determine antiplatelet and anti-inflammatory activity, against three agonists, PAF, ADP and thrombin. In summary, among the polyphenolic compounds, curcumin demonstrated the most potent inhibitory effect across all three metabolic pathways evaluated. The effects of curcumin appear to be consistent with Lee (2005), who also demonstrated the potent activity of the phenolic molecule against PAF [65]. Furthermore, according to Shah et al. (1999) [66], curcumin appears to be an extremely potent molecule against aggregation via the PAF, with an IC_50_ value of 25 μM. According to Balestrieri et al. (2003) [67], pre-incubation of platelets with 25 μM curcumin caused inhibition of PAF-induced aggregation. Maheswaraiah et al. (2015) [68], who investigated the inhibition of curcumin on ADP-induced aggregation, found an IC_50_ value of 135.74 μM (50 μg/mL). Τhe IC_50_ value was considerably higher than that of Rustichelli et al. (2024) [69], where the 50% inhibition of thrombin was found to be considerably lower at 24.09 μM.

Tannic acid showed a satisfactory inhibitory effect in the ADP assay, although its potency was significantly lower compared to curcumin and quercetin, the latter of which exhibited the highest potency in the same assay. Although tannin did not exhibit a strong inhibitory effect, its moderate capacity to inhibit platelet aggregation suggests potential for further investigation.

Within the flavonoid class, quercetin and catechin both showed effective inhibitory activity. Quercetin was particularly bioactive against thrombin, positioning it as a promising natural antiplatelet candidate. Catechin displayed consistent activity across all assays, with heightened sensitivity towards inhibiting PAF and thrombin, highlighting its potential as a natural therapeutic agent for inflammatory and thrombotic conditions. The inhibition of lyso-PAF was achieved by Yanoshita et al. [70], who found an IC_50_ value of 80 μM for quercetin, characterizing its action as quite strong. Also, Dianita and Jantan (2019) [71] in their study, where they isolated quercetin from leaf extracts and tested its action on human platelets against ADP-induced aggregation, found a value of 173.2 μM. Rodriguez et al. (2024) [72] also report the strong action of the flavonoid molecule, with an IC_50_ value of 2150.5 μM (0.65 mg/mL). In the research of Kang et al. (2002) [73], the inhibition of platelet aggregation by the action of catechin was studied, where the value found was IC_50_ = 1550.3 μM (0.45 mg/mL). Bijak et al. (2014) [74] found that thrombin inhibition by catechin was IC_50_ = 125 μM. These findings align with the observed efficacy of vitamin C and flavonoid supplementation.

Simple phenolic molecules, in contrast, exhibited the lowest inhibitory capacities across all three tested factors. Thymol exhibits the weakest inhibitory capacity, which agrees with the findings of Okazaki et al. (2002) [75]. The result for the action of gallic acid differs from that of Toyama et al. (2022) [76], who report in their findings the strong action of this acid with IC_50_ = 0.125 μM, where they showed that the compound interacts with the active site of platelet-activating factor acetylhydrolase (PAF-AH), inhibiting the hydrolysis of platelet-activating factor (PAF). These differences in IC_50_ values in our experimental study and in the literature may be due to the fact that we tested the value in human platelets, while the aforementioned study tested it in animal platelets. Rodriguez et al. (2024) [72], showed that the effect of gallic acid against ADP is quite weak, which agrees with our findings, having an IC_50_ value greater than 5,000 μM. The activity of gallic acid was found to be weak, contrasting with the findings of Zhang et al. (2022) [77] where they showed that this molecule is a fairly potent inhibitor of the thrombin factor in platelet aggregation with an IC_50_ value of 9.07 μM. Furthermore, the weak effect of vanillin agrees with Richardson et al. (2022) [78], who also showed no effect of this phenolic molecule on the total or specific phase of ADP-induced platelet aggregation.

Important results were revealed in this assay, enlightening intermolecular mechanisms with the enriched supplement, showing sensitivity in the PAF pathway and the establishment of the synergy of vitamin C and flavonoids, with this combination having the strongest activity compared to vitamin C-only supplement and phenolic standards. Vitamin C alone showed lower IC50 values against PAF-induced platelet aggregation compared to ADP-induced aggregation, suggesting a more pronounced inhibitory effect on the PAF pathway. The higher IC50 value of ADP indicates a comparatively weaker response, implying that a larger concentration of vitamin C was required to exert an equivalent inhibitory effect under ADP stimulation. This pattern suggests that vitamin C may preferentially inhibit PAF-mediated platelet activation, which aligns with its proposed role in modulating inflammatory pathways. This observation aligns with the hypothesis that vitamin C may preferentially modulate PAF-mediated platelet activation, potentially through its role in inflammatory pathway regulation. However, the lack of statistical significance and small effect sizes indicate that the observed differences could be due to variability rather than a consistent biological effect.

According to the bibliography [20], in research conducted with similar models investigating orange juice, fresh or oxidized, and comparing it with vitamin C-only dietary supplement, the IC_50_ values in terms of vitamin C against PAF were significantly higher than the values of the present supplement enriched with bioflavonoids. Specifically, the IC_50_ values of the vitamin C supplement almost reached 87 μg for fresh solution and 160 μg for the oxidized one, which are relatively higher than the values in the enriched supplement with a range 41–56 μg. Also, comparing the values of the enriched supplement with vitamin C only (72.7 μg), it showed much higher IC_50_ values, further supporting that there is synergy between the components. Last but not least, regarding the simple phenolics that were also examined, for example, quercetin (range 39, 36 μg) and catechin (range 28, 15 μg), these values were almost 10 times greater than the flavonoids in the enriched supplement, with IC_50_ values of 1.59 μg, showing the beneficial outcome in both components when combined together. Within the ADP-stimulated group, significant differences were observed between IC_50_ values, with bioflavonoids again having the lowest IC_50_ values. Likewise, the values calculated for the ADP pathway, were significant lower when compared to quercetin, catechin, or the standard phenolics, as shown in Table 4. Also, comparing the vitamin C-only supplement and the enriched supplement, the enriched supplement again has lower IC_50_ values, resulting in better efficacy, which is explained by the coexistence of the two compounds in the supplement. Considering all of the above led us to the conclusion that flavonoids have strong antiplatelet activity against the ADP pathway on their own although they are especially synergistic with vitamin C.

In vivo evaluation of the supplements revealed temporal changes in EC_50_ values for certain agonists, indicative of a time-dependent response regardless of the treatment group. Specifically, for both PAF and thrombin, participants exhibited significantly elevated EC_50_ values at Day 28 relative to baseline (Day 0), suggesting reduced platelet reactivity and supporting the supplements’ anti-inflammatory and antithrombotic properties. However, these changes were consistent across both the vitamin C alone (VC) and the vitamin C plus flavonoid (VCF) groups, with no significant differences in either the direction or magnitude of response attributable to treatment. Although visual inspection suggested a trend toward higher EC_50_ values for PAF at Day 28 in the VCF group, which highlights the potential of the VCF supplement to more effectively attenuate thrombo-inflammatory responses in vivo, possibly due to the synergistic contribution of flavonoids in enhancing the overall bioactivity of the supplement, this was not statistically significant.

For the ADP agonist, only minor temporal or intergroup differences were observed, with no clear pattern emerging. Moreover, the decrease in the EC_50_ value in the VC supplement may reflect the limited efficacy of the supplements in modulating platelet aggregation via this pathway and, in contrast, may highlight a possible pro-aggregatory effect. Overall, these findings suggest that platelet reactivity, as measured by EC_50_, changed over time for certain agonists, particularly PAF and thrombin, but that the addition of flavonoids to vitamin C supplementation did not significantly influence this trajectory compared to vitamin C alone. Last but not least, the non-significant difference of ADP is also an important result, ensuring safety of the supplements against hemolytic side effects and bleeding risk.

Altogether, these observations underscore the added value of flavonoids in the VCF formulation, enhancing the anti-PAF and antithrombin effects without amplifying the possibility of an ADP-related pro-aggregatory trend seen with the VC supplement alone. Further investigation is needed to confirm the statistical significance of these findings and to explore the therapeutic potential suggested by this research.

## 5. Conclusions

Our comprehensive evaluation of the vitamin C and bioflavonoid supplement elucidated its rich phytochemical profile and significant biological potential. The supplement exhibited a substantial total phenolic content, with flavonoids contributing prominently to its strong antioxidant capacity across multiple assays (DPPH, FRAP, ABTS) and suggesting synergistic mechanisms, with vitamin C potentially acting to stabilize and enhance the antioxidant activity of flavonoids. However, antioxidant activity varied by assay and compound, highlighting the complexity of phenolic interactions. The synergistic mechanisms were further noticed in the in vitro and ex vivo aggregometry assays, with VCF supplement showing superior activity against vitamin C-only supplement and phenolics. Notably, quercetin demonstrated superior reducing power and antiplatelet activity, identifying it as a key bioactive flavonoid, while catechin also showed consistent inhibitory effects on platelet aggregation and among polyphenols, such as curcumin. Both supplements showed promising in vitro biological activities, with statistically significant differences in efficacy. Simple phenolic compounds demonstrated comparatively lower inhibitory effects. The supplements showed increased sensitivity towards the PAF pathway, and insignificant results in the ADP pathway ensured a low bleeding risk. These findings underscore the therapeutic potential of bioflavonoids and polyphenols as natural antioxidants and antiplatelet agents in combination strategies with vitamin C, showing an enhanced activity of these two bioactive natural components, with very promising effects. Further investigation is required to optimize limitations. Overall, this study advances the understanding of natural compound interactions and supports their development as complementary agents in managing oxidative stress, inflammation, and thrombosis.

This study has certain limitations that must be considered in order to interpret the results correctly. Specifically, the statistical validity of the analyses is limited by the small number of healthy volunteers. In addition, our study was based on guidelines from the European Food Safety Authority (EFSA), who has proposed that a 4-week administration of a compound is sufficient to check for its antiplatelet cardioprotective potency. However, other researchers propose that 4 weeks is a short duration and that a clinical trial of this length does not allow for the observation of long-term effects of the substances we studied, while the relatively low dose administered, although based on commercially available formulations commonly available on the market for daily use, may affect the observation of systematic pharmacological effects. Furthermore, the absence of a control group receiving only flavonoids or a placebo limits the ability to isolate the specific effects of the intervention. Finally, variability in individual dietary habits and baseline antioxidant status, along with the lack of detailed plasma biomarker measurements, further limits the reliability and generalizability of the results.

## Figures and Tables

**Table 1:** Physicochemical properties of targeted phenolic bioactive compounds *.

Bioactives	Chemical Structure	Molecular Formula	Molecular Weight (g/mol)	pKa	Water Solubility (mg/L) 25 °C	logKow
Gallic acid	** 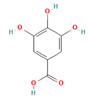 **	C_7_H_6_O_5_	170.12	4.40	12,000	0.7
Vanillin	** 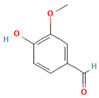 **	C_8_H_8_O_3_	152.15	7.7	10,000	1.2
Thymol	** 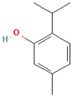 **	C_10_H_14_O	150.22	10.6	900	3.3
Quercetin	** 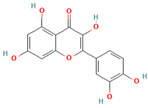 **	C_15_H_14_O_7_	302.23	6.38	2.15	1.5
Catechin	** 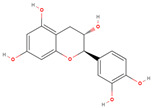 **	C_15_H_14_O_6_	290.27	9.00	450	0.4
Curcumin	** 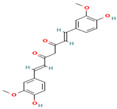 **	C_21_H_20_O_6_	368.4	7.7–8.5	3.12	3.2
Tannin	** 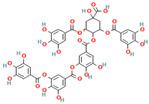 **	C_42_H_32_O_26_	952.7	~7	10,000–20,000	2.5
Tannic acid	** 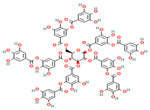 **	C_76_H_52_O_46_	1701.2	7.61	250,000	6.2

* physicochemical parameters were collected from PubChem (https://pubchem.ncbi.nlm.nih.gov, accessed on 28 April 2025).

**Table 2:** Comparative analysis of ATR-FTIR spectrum between phenolic standards and vitamin C and bioflavonoids supplement.

Wavenumber (cm^−1^)	Functional Group	Flavonoids Standards	Supplement with Vit C + Flavonoids	Explanation
3200–3600	O–H stretching	Present	Present	Hydrogen bonding, possible interaction between flavonoids and vitamin C
2900–2850	C–H aliphatic stretching	Weak but present	Present	Presence of organic components
1750–1680	C=O stretching (carbonyl)	Present	Present and shifted	Confirms presence of vitamin c and interaction with flavonoids
1650–1600	C=C aromatic ring	Strong	Enhanced peaks	Decreased transmittance potentially indicates aromatic flavonoids
1500–1400	C–C	Present	Different peaks	Present phenolic compounds
1300–1000	C–O stretching, C–H bending	Strong	Strong	Flavonoids presence
1270–1150	C–O–C glycosidic bonds	Strong	Present	Flavonoids presence

**Table 3:** Antioxidant activity of standard phenolics of the supplement containing solely vitamin C (VC) and of the supplement containing vitamin C with bioflavonoids (VCF), as quantified in vitro by the three different bioassays (DPPH, ABTS, and FRAP).

	DPPH (TEAC Values)	ABTS (ABTS Values: µmol TE/g)	FRAP (FRAP Values: µmol TE/g)
	Mean (±SD)	Mean (±SD)	Mean (±SD)
Simple Phenolics			
Gallic acid	0.00564 (±0.00023)	3,123,181.19 (±112,724.32)	31.41 (±2.99)
Vanillin	0.00161 (±0.00036)	8.48 (±1.87)	7.47(±7.29)
Thymol	0.00458 (±0.00069)	20,702.18 (±1772.83)	6.10(±5.07)
Flavonoids			
Quercetin	0.00226 (±0.00183)	45,051.76 (±25,238.42)	2274.30 (±124.49)
Catechin	0.00543 (±0.00007)	14.00 (±0.08)	2448 (±20)
Polyphenols			
Curcumin	0.00124 (±0.00047)	1033.57 (±332.90)	197.83 (±50.82)
Tannin	0.00544 (±0.00026)	6651.88 (±6069.30)	27.32 (±1.25)
Tannic acid	0.00546 (±0.00043)	20.89 (±21.46)	19.80 (±5.81)
VC supplement			
Vitamin C	3.48300 (±2.30333)	245,419.82 (±276,843.91)	3795.45 (±3346.02)
VCF supplement			
Vitamin C	3.35360 (±0.65361)	298,268.60 (±290,782.80)	4137.89 (±2806.14)
Flavonoids	6.70720 (±1.30721)	596,537.20 (±581,565.70)	8275.78 (±5612.27)

**Table 4:** Descriptive statistics analysis of phenolic supplements in vitro with PAF, ADP, and thrombin inhibitor in μM and μg.

	PAF			ADP			THROMBIN			
Compound	Mean (μΜ) (±SD)		Mean (μg) (±SD)	Mean (μΜ) (±SD)		Mean (μg) (±SD)	Mean (μΜ) (±SD)		Mean (μg) (±SD)	
Simple Phenolics										
Gallic acid	11,152.87 (±4326.21)		474.33 (±183.99)	6419.51 (±1479.5)		273.02 (±62.92)	2718.67 (±383.86)		115.62 (±16.32)	
Vanillin	4620.75 (±2745.14)		175.76 (±104.42)	2298.36 (±838.26)		87.42 (±31.88)	2738.74 (±1453.9)		104.17 (±55.30)	
Thymol	5734.80 (±4303.92)		215.37 (±161.63)	7615.95 (±2976.6)		286.02 (±111.79)	9200.53 (±3134.9)		345.52 (±117.73)	
Flavonoids										
Quercetin	520.88 (±314.95)		39.36 (±23.80)	147.89 (±55.41)		11.17 (±4.19)	128.97 (±30.34)		9.74 (±2.29)	
Catechin	387.99 (±205.86)		28.15 (±14.94)	4163.24 (±2476.15)		302.10 (±179.68)	145.62 (±22.83)		10.57 (±1.66)	
Polyphenols										
Curcumin	209.36 (±42.29)		19.28 (±3.89)	135.17 (±40.67)		12.45 (±3.75)	140.61 (±86.51)		12.95 (±7.97)	
Tannin	712.91 (±446.07)		300.20 (±189.71)	433.56 (±194.15)		184.40 (±82.57)	478.09 (±156.00)		203.33 (±66,35)	
Tannic acid	598.31 (±43.88)		254.46 (±18.65)	283.54 (±263.32)		120.59 (±111.99)	614.18 (±243.08)		261.21 (±103.38)	
VC Supplement										
Vitamin C	1651.06 (±544.24)		72.70 (±23.96)	2287.63 (±1493.9)		100.72 (±65.78)				
VCF Supplement			33.36 (±14.53)			538.3 (±217.39)				
Vitamin C			31.77 (±13.84)			512.67 (±207.04)				
Flavonoids			1.59 (±0.69)			25.63 (±10.35)				

**Table 5:** Accumulative results of the in vivo evaluation of the anti-inflammatory (anti-PAF)/antiplatelet (anti-ADP)/antithrombotic (antithrombin) health-promoting properties of the VC and VCF supplements prior to and after 28 days of administration (data are expressed as EC_50_ values for each thrombo-inflammatory agonist).

EC_50_ of Each Agonist	Day	Group	N	Mean (±SD)	Median	Min	Max
PAF EC_50_ (nM)	Day 0	VC	7	12.94 (±6.33)	9.85	6.34	20.62
	Day 0	VCF	8	11.79 (±5.51)	9.99	5.24	21.09
	Day 28	VC	7	14.96 (±4.65)	14.10	7.75	21.98
	Day 28	VCF	8	20.14 (±8.20)	20.05	7.89	34.68
ADP EC_50_ (μM)	Day 0	VC	7	9.47 (±4.59)	10.26	1.88	14.57
	Day 0	VCF	8	13.91 (±8.95)	12.55	3.85	31.64
	Day 28	VC	7	5.84 (±4.89)	4.05	1.52	15.03
	Day 28	VCF	8	10.62 (±5.37)	11.45	3.21	17.05
THROMBIN (TRAP) EC_50_ (μM)	Day 0	VC	7	30.30 (±12.68)	25.93	13.41	52.76
	Day 0	VCF	7	32.16 (±9.67)	31.90	19.21	47.97
	Day 28	VC	7	57.17 (±16.47)	59.71	28.92	81.08
	Day 28	VCF	8	44.10 (±12.63)	43.68	23.00	66.00

## Data Availability

All data are contained within the article. Any further information can be provided by the authors upon request.

## Supplementary Materials

The following supporting information can be downloaded at: https://www.mdpi.com/article/10.3390/nu17162643/s1, Figures S1–S9: (Supplementary Figure S1. Gallic acid FTIR spectrum, Supplementary Figure S2. Vanillin FTIR spectrum, Supplementary Figure S3. Thymol FTIR spectrum, Supplementary Figure S4. Quercetin FTIR spectrum, Supplementary Figure S5. Catechin FTIR spectrum, Supplementary Figure S6. Curcumin FTIR spectrum, Supplementary Figure S7. Tannin FTIR spectrum, Supplementary Figure S8. Tannic acid FTIR spectrum, Supplementary Figure S9. Ascorbic acid standard spectrum).
