# Supplementary material for: Anti-Inflammatory, Antithrombotic and Antioxidant Efficacy and Synergy of a High-Dose Vitamin C Supplement Enriched with a Low Dose of Bioflavonoids; In Vitro Assessment and In Vivo Evaluation Through a Clinical Study in Healthy Subjects
Source: Nutrients. 2025 Aug 14;17(16):2643. doi: 10.3390/nu17162643 (PMC12389177; doi:10.3390/nu17162643)
Supplement: Supplementary file 1 [file nutrients-17-02643-s001.zip › nutrients-3754440-supplementary.pdf]

Supplementary ART-FTIR obtained spectrum of standard phenolic compounds

S1 Simple Phenolics

<u>S1.1. Gallic acid</u>

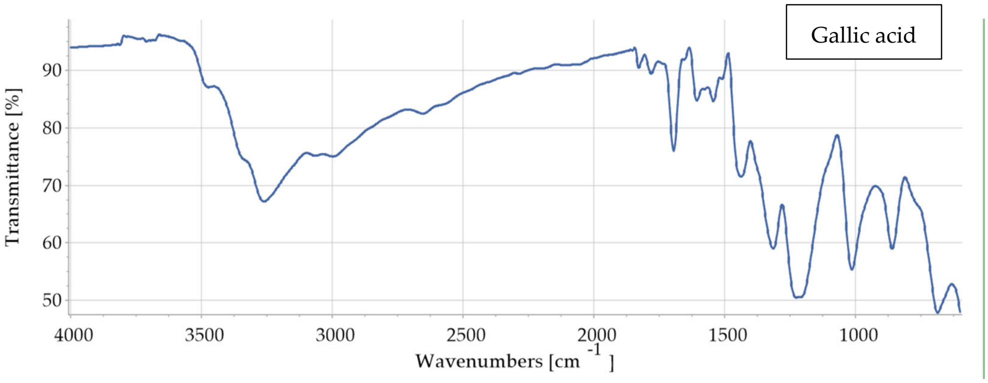

Supplementary Figure 1. Gallic acid FTIR spectrum

In the spectrum of gallic acid (S1), an intense peak is observed around 3300 cm^{-1} , assigned to the stretching vibration of the hydroxyl group -OH, while the peak around 1700 cm^{-1} indicates the presence of the carbonyl group C=O in the molecule. The peaks appearing at 1220 cm^{-1} and 1010 cm^{-1} are assigned to stretching vibrations of the C-O bond and bending vibrations of the O-H bond of the acid.

<u>S1.2. Vanillin</u>

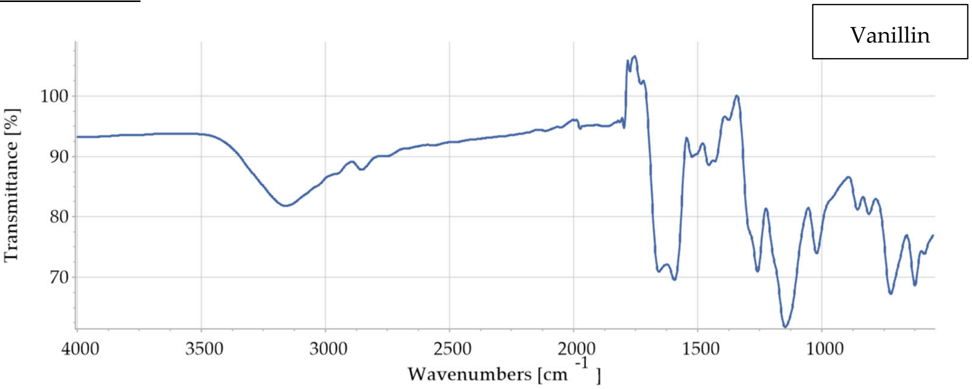

Supplementary Figure 2. Vanillin FTIR spectrum

The spectrum of pure vanillin powder (S2) , exhibits an intense peak around 3100 cm^{-1} , revealing OH stretching and bending vibration group.Also , we can observe three more peaks at 1590 cm^{-1} , at 1450 cm^{-1} and 720 cm^{-1} , which all correspond to the absorption of stretching vibrations of the benzene ring for the C=C bonds. Finally, the absorption peak at 1150 cm^{-1} indicates the presence of ether groups in the molecule.

<u>S1.3. Thymol</u>

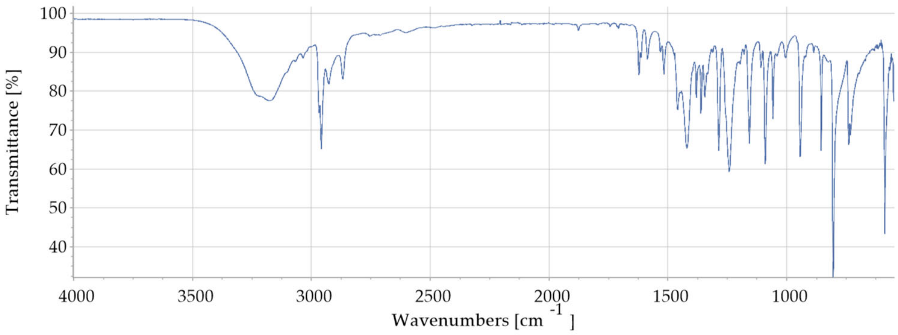

Supplementary Figure 3: Thymol FTIR spectrum

In the spectrum of thymol we observe a characteristic peak near 3200 cm^{-1}, corresponding to the hydroxyl group -OH. In addition, a peak at 2960 cm^{-1} represents the sp3 bond of C-H. The peak revealing the aromaticity of the molecule is located near 1600 cm^{-1} , with a weak peak indicating the C=C bonds of the benzene ring.

S2. Flavonoids

S.2.1. Quercetin

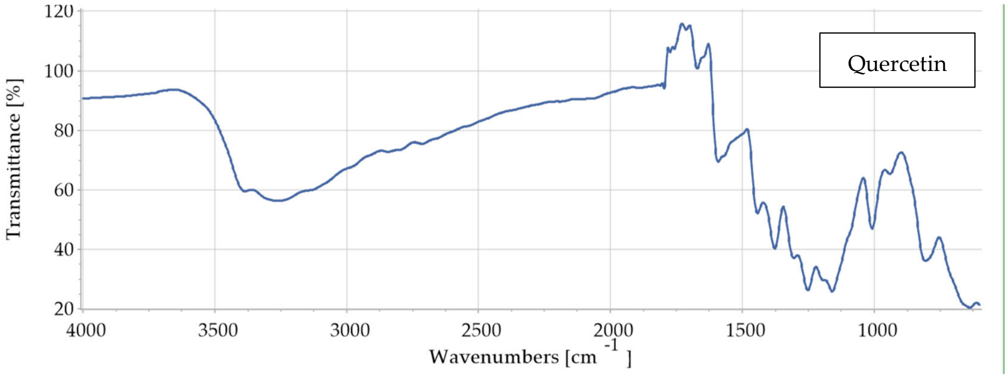

Supplementary Figure 4. Quercetin FTIR spectrum

As shown in the FTIR spectrum of quercetin (S4), a weak peak around 3300 cm^{-1} characterizes the hydroxyl group in the molecule.Moreover, the peak around 1600 cm^{-1} indicates the stretching of the aromatic ring C=C, while the absorption at 1250 cm^{-1} and at 1160 cm^{-1} are assigned to the C–O stretching in phenol and the C–CO–C stretch and bend in ketone.

S.2.2. Catechin

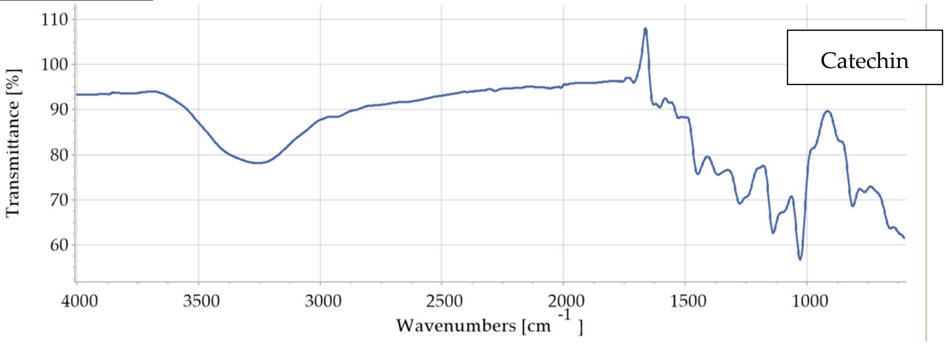

Supplementary Figure 5. Catechin FTIR spectrum

As regarding catechin spectra (S5), the absorption at 3250 cm^{-1} corresponds to the hydroxyl group -OH , a characteristic group in the molecule of catechin.

Furthermore, the absorption around 1440 cm^{-1} indicates the presence of phenyl ring. Also, the absorption peaks at 1275 cm^{-1} and at 1140 cm^{-1} are originated from the C–O stretching vibration of molecule, and the absorption at 1030 cm^{-1} indicates an ether group.

S3. Polyphenols

S3.1. Curcumin

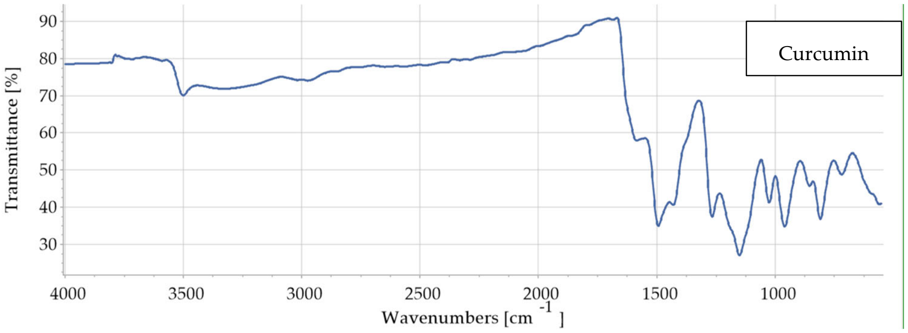

Supplementary Figure 6. Curcumin FTIR spectrum

As we can observe in curcumin spectra (S6) the weak peak appearing at 3500 cm^{-1} is due to the stretching of the hydroxyl O-H bonds, indicating the presence of free O-H bonds in the methoxyphenyl rings of the molecule. The peak around 1500 cm^{-1} is assigned to mixed vibrations, including the carbonyl bond stretching vibrations ν(C=O) while the peak at 1150 cm^{-1} is due to the bending vibration of the ν(C-O) phenolic band.

S3.2.Tannin

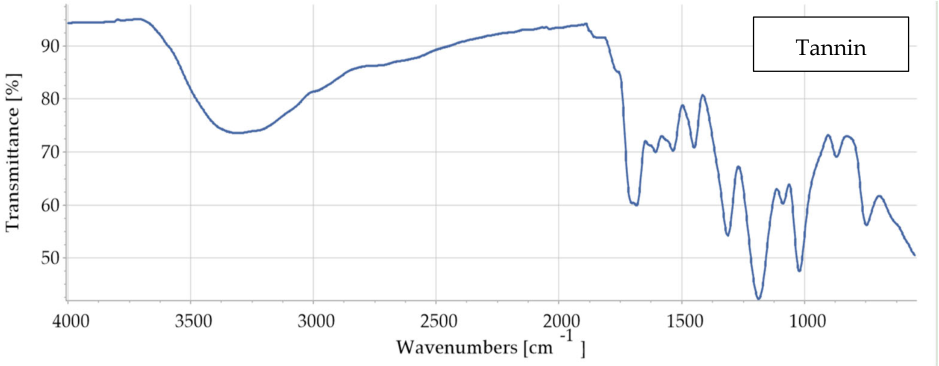

Supplementary Figure 7. Tannin FTIR spectrum

In S7 , according to Tannin FTIR spectrum, the broad peak at 3300 cm^{-1} indicates the presence of the hydroxyl group O-H in the molecule. Around 1680 cm^{-1}, the absorption is related to the presence of carbonyl C=O which is mainly found in hydrolysable tannins.Furthermore, the peak near 1300 cm^{-1} represents the C-H deformation region while the peaks around 1200 cm^{-1} and 1020 cm^{-1} correspond to the C–O stretching vibration.

S.3.3. Tannic acid

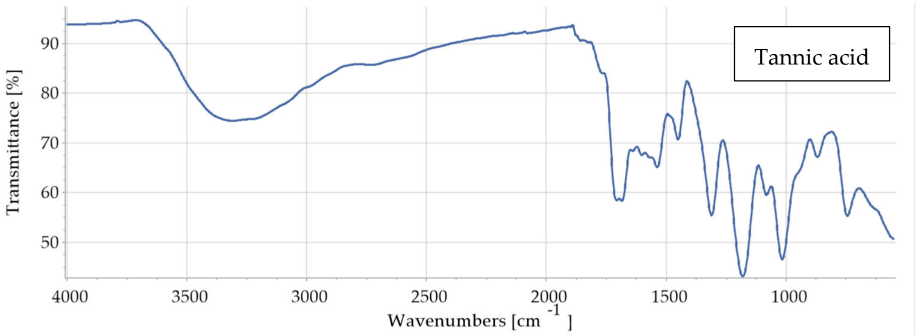

Supplementary Figure 8. Tannic acid FTIR spectrum

Observing the spectrum of tannic acid (S8), the peak of the spectrum which located around 3300 cm^{-1} is a broad peak, which is assigned to the hydroxyl groups (O-H) of the molecule. The absorption near 2700 cm^{-1}, although it is a weak peak in the spectrum, could represent the symmetric and asymmetric C-H stretching vibrations. The peak at 1660 cm^{-1}, reveals the existence of aromatic esters due to the characteristic signal of the carbonyl groups C=O at this specific absorption length, while the peak at 1180 cm^{-1} corresponds to the C-O bond.

S4. Ascorbic acid

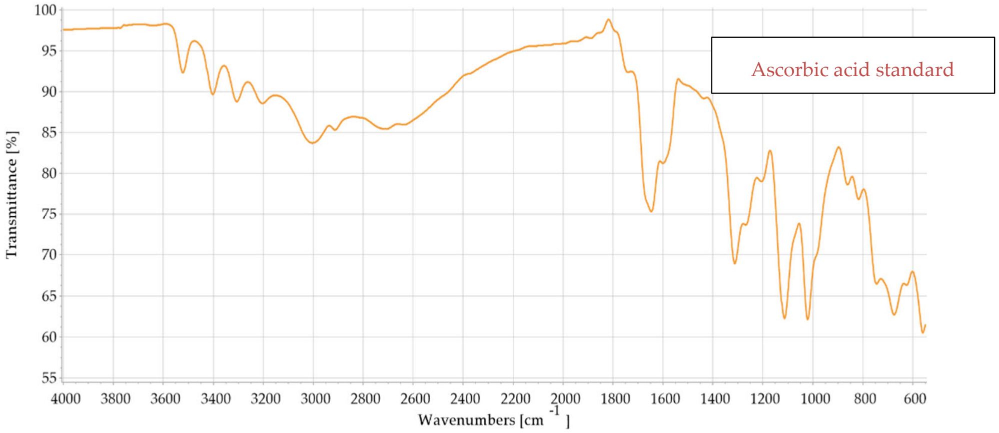

Supplementary Figure 9. Ascorbic acid standard spectrum
